# Wfs1 is expressed in dopaminoceptive regions of the amniote brain and modulates levels of D1-like receptors

**DOI:** 10.1371/journal.pone.0172825

**Published:** 2017-03-07

**Authors:** Triin Tekko, Triin Lakspere, Anni Allikalt, Jaanus End, Karl Rene Kõlvart, Toomas Jagomäe, Anton Terasmaa, Mari-Anne Philips, Tanel Visnapuu, Fred Väärtnõu, Scott F. Gilbert, Ago Rinken, Eero Vasar, Kersti Lilleväli

**Affiliations:** 1 Department of Physiology, Institute of Biomedicine and Translational Medicine, University of Tartu, Tartu, Estonia; 2 Centre of Excellence in Genomics and Translational Medicine, University of Tartu, Tartu, Estonia; 3 Department of Developmental Biology, Institute of Molecular and Cell Biology, University of Tartu, Tartu, Estonia; 4 Institute of Chemistry, University of Tartu, Tartu, Estonia; 5 Department of Biology, Swarthmore College, Swarthmore, PA, United States of America; University Paris Diderot, FRANCE

## Abstract

During amniote evolution, the construction of the forebrain has diverged across different lineages, and accompanying the structural changes, functional diversification of the homologous brain regions has occurred. This can be assessed by studying the expression patterns of marker genes that are relevant in particular functional circuits. In all vertebrates, the dopaminergic system is responsible for the behavioral responses to environmental stimuli. Here we show that the brain regions that receive dopaminergic input through dopamine receptor D_1_ are relatively conserved, but with some important variations between three evolutionarily distant vertebrate lines–house mouse (*Mus musculus*), domestic chick (*Gallus gallus domesticus*) / common quail (*Coturnix coturnix*) and red-eared slider turtle (*Trachemys scripta*). Moreover, we find that in almost all instances, those brain regions expressing D1-like dopamine receptor genes also express *Wfs1*. Wfs1 has been studied primarily in the pancreas, where it regulates the endoplasmic reticulum (ER) stress response, cellular Ca^2+^ homeostasis, and insulin production and secretion. Using radioligand binding assays in wild type and *Wfs1*^-/-^ mouse brains, we show that the number of binding sites of D1-like dopamine receptors is increased in the hippocampus of the mutant mice. We propose that the functional link between Wfs1 and D1-like dopamine receptors is evolutionarily conserved and plays an important role in adjusting behavioral reactions to environmental stimuli.

## Introduction

The *Wfs1* gene encodes wolframin, an ER-resident membrane protein whose functions include the regulation of insulin production and secretion from pancreatic β-cells, as well as the regulation of ER stress response, cellular Ca^2+^ homeostasis, and secretory granule acidification [1–14]. In humans, loss of functional WFS1 protein results in Wolfram syndrome, characterized by diabetes insipidus, diabetes mellitus, optic atrophy and progressive sensorineural deafness often accompanied by psychiatric and neurological symptoms [15–18]. The mechanisms underlying the disturbances in the appropriate functioning of the brain are largely unknown.

We and others have previously shown that Wfs1 is enriched in those regions of the rodent brain associated with the control of behaviours and emotions, and with the relay of sensory and motor signals: layer II/III of the cerebral cortex, the CA1 field of the hippocampus, the central extended amygdala, the ventral and dorsal striatum, and various sensory and motor nuclei of the brainstem [19–22]. Functional studies demonstrate that Wfs1 is critical for normal dopamine secretion in the striatum and for dopamine transporter expression in the midbrain [23–24]. Moreover, *Wfs1*-deficient mice display abnormal responses to dopamine agonists [24–25].

Since the dopamine system is altered in *Wfs1*-deficient mice, and Wfs1 has been shown to regulate cyclic AMP synthesis in pancreatic β-cells [11], we hypothesized that Wfs1 may be involved in D1-like dopamine receptor signalling, which is also positively coupled to cyclic AMP synthesis [26]. Therefore, we studied the expression of D1-like receptors in parallel with Wfs1 and examined whether D1-like receptor-specific ligand binding is altered in the hippocampi of *Wfs1*^-/-^ mice.

In addition, we were interested in whether homologous brain structures in different amniote lineages (as defined by marker gene expression and neural connectivity) also reflect functional similarities. Since *Wfs1* expression defines discrete structures in rodent brain, we determined whether the brain regions receiving dopaminergic input through dopamine receptor D_1_ also express *Wfs1* in two other vertebrate lines: the domestic chick / the common quail and the red-eared slider turtle.

## Materials and methods

### Animals

Brains of mouse (*Mus musculus*), n = 4 for *in situ* hybridization and immunohistochemistry, n = 82 for radioligand binding assay; chicken (*Gallus gallus domesticus*), n = 6; common quail (*Coturnix coturnix*), n = 2; and red-eared slider turtle (*Trachemys scripta*), n = 2, were used in this study. Wild-type C57BL/6 (Scanbur, Karlslunde, Denmark), and *Wfs1*^*-/-*^ mice were housed under standard laboratory conditions (12-h light/dark cycle with free access to food and water) at the Laboratory Animal Centre of the Institute of Biomedicine and Translational Medicine, University of Tartu (accreditation number KL1210), and were killed by cervical dislocation and, in case of transcardial perfusion, anaesthetized by overdose approved by the Estonian National Board of Animal Experiments. *Wfs1*^*-/-*^ mice do not suffer from gene inactivation, studies with *Wfs1*^*-/-*^ mice have been approved by the Estonian National Board of Animal Experiments (No. 86, 28.08.2007) and are in accordance with the European Union directive 86/609/EEC.

Obtaining animal tissues was performed after rapid execution, no manipulation with the animals occurred before. Thus, according to European Union directive 2010/63/EU Article 3, the activities performed in the current study cannot be considered animal experimentation.

Adult chick brains were obtained from commercial poultry farming company Tallegg (license nr 25 from the Veterinary and Food Board of Estonia), and the embryonic and newly hatched chicks were obtained from the Science Centre AHHAA in Tartu, Estonia in cooperation with Tallegg.

Quail brains were obtained from commercial quail farming company Järveotsa Vutifarm OÜ (license nr 40 from the Veterinary and Food Board of Estonia).

Turtles were purchased commercially from the Kliebert Turtle and Alligator Farm (Hammond, Louisiana) and were killed under anesthesia and cold according to protocols approved by Swarthmore College IACUC committee #07-9-20.

### Tissue preparation

All mice used in the experiments were killed by cervical dislocation and chicken and quails by decapitation. Decapitation of turtles was performed under ketamine and xylazine anaesthesia (90 mg/kg and 6 mg/kg, respectively) combined with hypothermia induced by keeping the animal in ice. The anaesthetic was injected intramuscularly into the front limb muscle.

In case of chick embryos, embryonic day 0 (E0) was designated as the day when the egg was transferred to 37°C. For *in situ* hybridization, brains were fixed with 4% PFA/PBS for 5 days at +4°C, after which the brains were cryoprotected overnight with 20% sucrose in 4% PFA/PBS at +4°C and stored at −80°C until sectioning. For immunohistochemical experiments, adult mice were anaesthetised with intraperitoneal injection of ketamine-xylazine (100 mg/kg and 10 mg/kg, respectively) through the right side of the abdominal wall. Subsequently, transcardial perfusion was performed with PBS followed by 2% PFA/PBS. The brains were dissected and kept in 2% PFA/PBS for 1 h and cryoprotected overnight in 20% sucrose in 1% PFA/PBS at +4°C. For fluorescent immunohistochemistry quail and turtle brains were fixed for 4 h in 4% PFA/PBS, washed with PBS following impregnation with 30% sucrose in milli-Q at +4°C, and were frozen and stored at −80°C. For radioligand binding experiment, the hippocampi were dissected on ice immediately after decapitation, frozen in liquid nitrogen and stored at -80°C until further processing.

### *In situ* RNA hybridization

The non-radioactive *in situ* hybridization was carried out as described in [22]. The mouse *Wfs1* riboprobe was the same as in [22]. cDNA fragment sequences used as templates for Dig-labelled riboprobes for other genes were obtained using the following primers (containing NotI and SalI restrictase sites):

**Table:** 

Mouse *Drd1a* For	TTTGC ˘GGCC̭GCctctgctgcttttggacag
Mouse *Drd1a* Rev	TTTG ˘TCGA̭Ctaggggcagagcattggtag
Mouse *Drd5* For	TTTGC ˘GGCC̭GCgagaactgtgactccagcct
Mouse *Drd5* Rev	TTTG ˘TCGA̭Cgacatgtgatcgaaaggccc
Chick *Drd1a* For	TTTGC ˘GGCC̭GCatgacttggaacgacaccact
Chick *Drd1a* Rev	TTTG ˘TCGA̭Cagttgctctcaggttgctgg
Chick *Wfs1* For	TTTGC ˘GGCC̭GCgacagaagaggcatcacttctgagaa
Chick *Wfs1* Rev	TTTG ˘TCGA̭Cctcatgtagcttgtcactgtgaagaa

In case of turtle, we used chick *Wfs1* and *Drd1a* probes and the hybridization was carried out at 60°C and post-hybridization washes at 60°C and 57°C. Using NCBI BLAST, we analyzed the sequence identity between the chick cDNA sequences corresponding to the probes and the transcriptome of *Trachemys scripta* (accessible from [27]. The sequence identity was 88% (682 nucleotides of 773) in case of *Drd1a* and 89% (786 nucleotides of 887) in case of *Wfs1*.

### Immunohistochemistry

Immunohistochemistry was performed on 40 μm freely floating coronal cryosections of adult mouse brain and all the steps were carried out under shaking conditions. After cutting, the sections were washed in PBS/0.25% TritonX-100 for 15 min. To quench the endogenous peroxidase, the sections were treated with 0.3% hydrogen peroxide in milli-Q for 15 min following three washes with PBS/0.25% TritonX-100. The sections were blocked for 1h in PBS/0.25% TritonX-100 containing 5% horse serum (Vector Laboratories) and incubated in PBS/0.25% TritonX-100 with 2% horse serum and primary antibody diluted in 1:1000. Rabbit polyclonal D_1_ (#ADR-001) and D_5_ (#ADR-005) antibodies were obtained from Alomone Labs. Rabbit polyclonal Wfs1 antibody was the same as in [20] (referred to as Wfs1C). The antibody binding was detected using the Vectastain Elite ABC Kit (Vector Laboratories) according to the protocol provided by the manufacturer. Briefly, the biotinylated secondary antibody combined with horseradish peroxidase reaction with DAB (Vector Laboratories) was used to visualize immunoreactivity.

For fluorescent immunohistochemistry, 40 μm freely floating quail and turtle coronal cryosections were permeabilized with 0.3% TritonX-100 / PBS over 30 min and blocked with 5% donkey serum (Jackson ImmunoResearch Laboratories Inc.) /1% BSA (Sigma) /PBS over 1 h with gentle rocking. Wfs1 antibody (for details, see above) dilution 1:400 in 1% BSA / 0.1% Tween-20 / PBS was applied and incubated over 1 h at RT, followed by overnight incubation at 4°C. Incubation in FITC conjugated goat anti-rabbit secondary antibody solution (1:1000, Jackson ImmunoResearch Laboratories Inc.) in 0.1% Tween-20 / 1% BSA/PBS was performed at room temperature over 2 h. Nuclei were counterstained with DAPI (4,6-diamidino-2-phenylindole, Sigma Aldrich) 1: 2000 dilution in secondary antibody buffer. Sections were further washed in PBS and mounted in Fluoromount (Sigma Aldrich) mounting medium. Specifity of the immunohistochemistry was determined by incubations without the Wfs1 primary antibody.

### Evaluation of expression signals

As we have previously shown, visual observation of relative gene expression obtained by the enzymatic *in situ* reaction correlates with the results obtained by using AutoQuantX3 software [22]. Accordingly, we categorized the expression levels as high (+++) when there was a relatively rapidly appearing and strong signal, compared to a moderate (++) stable signal. Low signals (+) were those detectable by microscopic evaluation, but not always unambiguously detectable in the images. We examined sequential sections throughout the brain from at least two individuals of each species.

### Imaging and analysis

Photomicrographs of *in situ* hybridized and immunostained sections were recorded using Olympus BX61 microscope with Olympus DX70 CCD camera (Olympus, Hamburg, Germany). Immuno-fluorescence images were taken with Olympus FV-1000 (Olympus) confocal microscope and processed with Adobe Photoshop CC (Adobe Systems Incorporated).

The chick/quail brain regions were determined according to [28], the turtle brain regions according to [29], and mouse brain regions according to [30].

### Radioligand [^3^H]SCH23390 binding assay

Hippocampal membranes were prepared as described earlier [31] with slight modifications. Hippocampi from wt and *Wfs1* knockout mice were homogenized in 1 ml of ice cold homogenization buffer (HB: 50 mM Tris-HCl, pH 7.4) with a Bandelin Sonoplus sonicator (Bandelin Electronic GmbH) for three 10 s cycles. Membrane suspensions were then centrifuged at 30 000 g for 20 minutes at 4°C. The membrane pellet was washed by resuspending in 1 ml of HB followed by three centrifugations. Final homogenization was done in 50 volume (ww/v) of incubation buffer (IB: 50 mM Tris-HCl, 120 mM NaCl, 5 mM KCl, 5 mM MgCl_2_, 1 mM EDTA, pH 7.4) with final concentration of 20 mg tissue/ml. The samples were stored at -90°C until further use.

All radioligand binding experiments were performed in 96-well plates, and the reactions were carried out in a final volume of 250 μl per well as described in [32] with some modifications. Assay buffer IB was supplemented with 1 mM of DTT immediately before the experiment. In radioligand binding curve experiments, the hippocampal membranes of 6 mice from corresponding group were pooled and used at concentration of 20 mg tissue/ml. The membranes were incubated with different dilutions of a radioligand [^3^H]SCH23390 (0.06 – 8.2 nM) in the absence (for total binding) or in the presence (for nonspecific binding) of 10 μM (+)-butaclamole, a dopaminergic antagonist. [^3^H]SCH23390 (81.9 Ci/mmol) was from PerkinElmer, (+)-butaclamole was purchased from Sigma-Aldrich. Samples were then incubated for 60 min at 25°C and the reactions were stopped by rapid filtration through thick GF/B glass fibre filtermats using FilterMate Harvester (both from PerkinElmer). Filters were then washed 5 times with ice-cold washing buffer (WB: 20 mM K-phosphate, 100 mM NaCl, pH 7.4), after which the filters were dried in a microwave oven at 800 W for 2 min. Solid scintillant MeltiLex^TM^ B/HS was then impregnated into the filter by using MeltiLex^TM^ Heatsealer. The filter-bound radioactivity was counted using a Wallac MicroBeta TriLux 1450 LSC Luminescence Counter (all from PerkinElmer). The total concentrations of radioligand dilutions were determined in vials with 3 ml of liquid scintillation cocktail OptiPhase HiSafe (PerkinElmer).

The number of binding sites of D1-like receptors in wt and *Wfs1* knockout mice was estimated by determination of specific binding of 4 nM [^3^H]SCH23390 to corresponding membrane preparation as described above. The tissue concentration in these experiments was 6.7 mg/ml.

All the data were analyzed using GraphPad Prism 5.0 (GraphPad Software Inc). Data are presented as mean ± SEM of at least three independent experiments carried out at least in duplicates. Statistically significant differences were determined by the Student test, where p < 0.05 was taken as the criterion of significance.

## Results

### Comparison of the expression of *Wfs1* with *Drd1a* and *Drd5* in mouse brain

#### mRNA distribution

**Fig 1:**
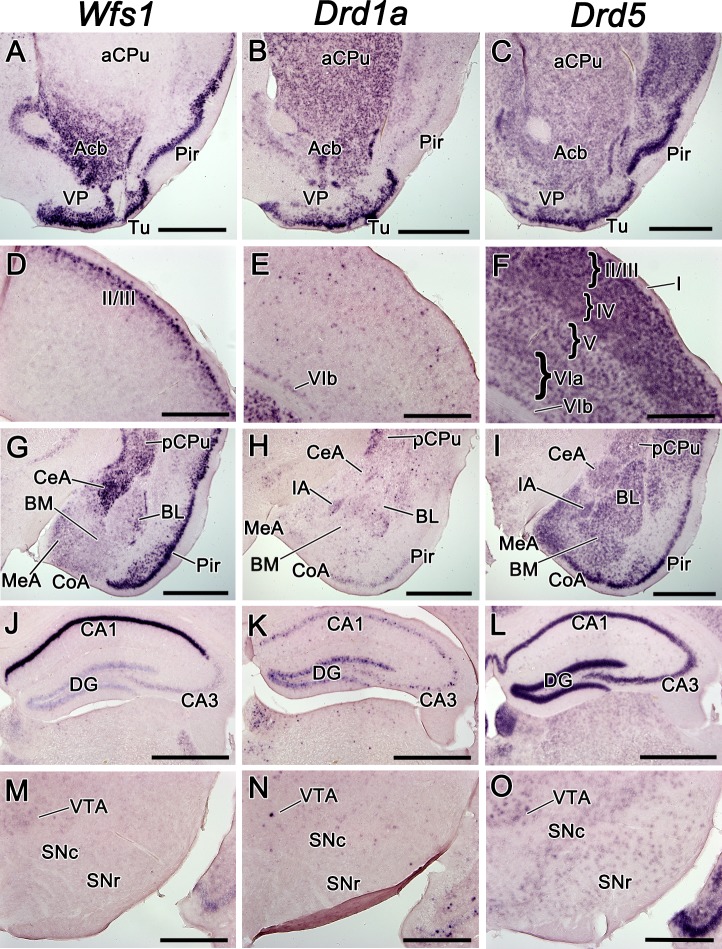
The mRNA expression pattern of *Wfs1*, *Drd1a*, and *Drd5* in the adult mouse brain. The mRNA expression pattern of *Wfs1*, *Drd1a*, and *Drd5* in the adult mouse brain, shown by in situ hybridization. In this and all subsequent figures, the medial side of the coronal sections is on the left and the lateral side on the right. The probes are indicated above. The expression in CPu and ventral striatum (A-C), in somatosensory cortex at the level of bregma 0.74 (D-F), in the amygdala at the level of central and basolateral nuclei (G-I), in the hippocampus (J-L), in the substantia nigra and VTA (M-O). For abbreviations, see list. Scale bar is 1 mm.

**Fig 2:**
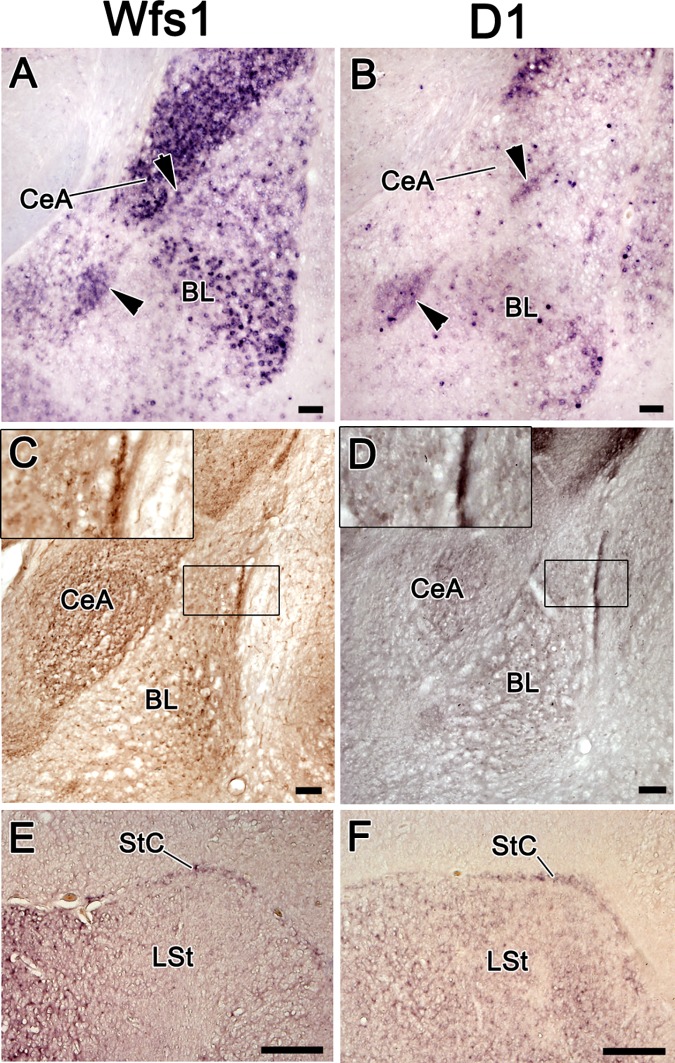
The expression of *Wfs1* and *Drd1a* in the mouse intercalated amygdala and in its putative avian homologue, StC, in chick. The expression of *Wfs1* and *Drd1a* in the mouse intercalated amygdala and in its putative avian homologue, StC, in chick. A, B–in situ hybridization on coronal sections of the mouse brain. C, D–immunohistochemistry on coronal sections of the mouse brain. E, F–in situ hybridization on the coronal sections of the chick brain. The intercalated nuclei of the amygdala (arrowheads) are expressing both Wfs1 and Drd1a in mouse brain (A, B). Wfs1 and D1 proteins are both strongly expressed in the intercalated nuclei (C, D). The insets in C and D show closer view on the intercalated nucleus between the BL and claustrum-endopiriform formation. In chick brain, the StC is expressing both *Wfs1* and *Drd1a*. For abbreviations, see list. Scale bar is 100 μm in A-D and 1 mm in E-F.

The transcription of *Wfs1*, *Drd1a*, and *Drd5* in the adult mouse brain showed specific regions of overlap between the mRNA expression domains of *Wfs1* and D1-like dopamine receptors. *Wfs1* expression overlapped with both *Drd1a* and *Drd5* in nucleus accumbens (Acb), olfactory tubercle (Tu), and posterior caudate-putamen (CPu; Fig 1A, 1B, 1C, 1G and 1H, 1I). In the cerebral cortex, *Wfs1* showed overlapping expression domain with *Drd5* in layer II/III of the neocortex and and in piriform cortex (Fig 1A, 1C, 1D, 1F, 1G and 1I). In the hippocampus, *Drd1a* and *Drd5* were expressed in all regions including CA1, CA3 and dentate gyrus (DG), sharing a common expression domain with *Wfs1* in CA1 (Fig 1J, 1K and 1L). In the amygdala, *Wfs1*, *Drd1a* and *Drd5* were coexpressed in all nuclei with varying expression levels: *Wfs1* was strong in the central nucleus of the amygdala (CeA), whereas *Drd1a* was present very weakly and *Drd5* at a moderate level; in the basolateral amygdala (BL), *Wfs1* showed a centrolaterally increasing expression gradient which was not present in case of *Drd1a* and *Drd5* (Fig 1G, 1H and 1I). The intercalated amygdala (IA) was delineated by the expressions of all three of these genes (Fig 1H and 1I; Fig 2A and 2B). In the substantia nigra, which is the source of dopaminergic fibres terminating in the CPu, *Wfs1* signal was not detectable (Fig 1M). A few sparse cells expressing *Drd1a* were observed in the pars compacta of the substantia nigra (SNc; Fig 1N). *Drd5* mRNA was moderately present in SNc and weakly in pars reticulata (SNr; Fig 1O). In the ventral tegmental area (VTA), another source of dopaminergic fibres that terminate in the ventral striatum and frontal cortex, we detected weak diffuse expression of *Wfs1*, the sparse cells expressing *Drd1a* and stronger diffuse expression of *Drd5* (Fig 1M, 1N and 1O).

#### Protein distribution

**Fig 3:**
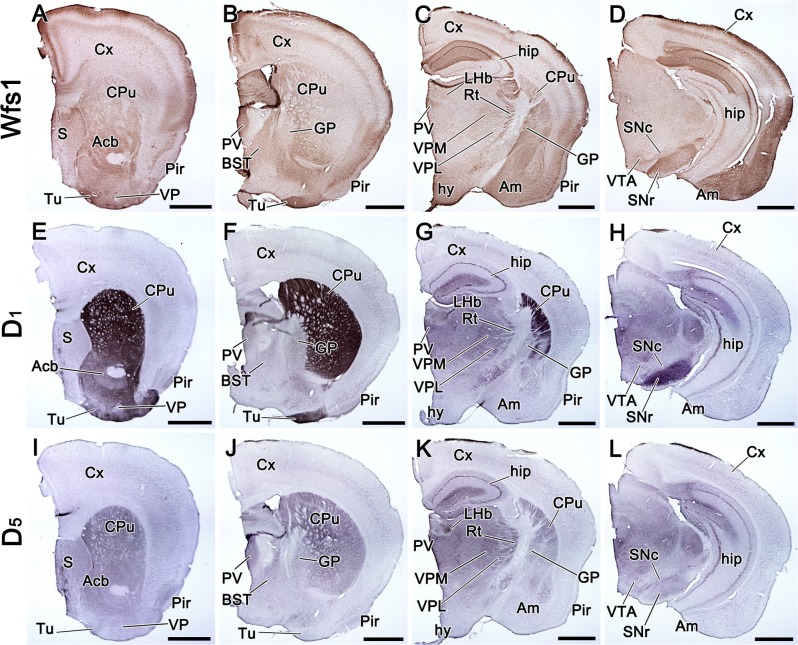
Distribution of Wfs1, D_1_, and D_5_ proteins in the adult mouse brain. Distribution of Wfs1, D1, and D5 proteins in the adult mouse brain, shown by immunohistochemistry on coronal sections. The sections are in anterio-posterior order from left to right. The detected proteins are indicated on the left side of the figure. Wfs1 is present in the cerebral cortex, in CA1 of hippocampus, CPu, Acb, Tu, amygdala, Rt, PV, VPM, hypothalamus and SNr (A-D). D1 is strongly present in CPu, Acb, Tu, hip, thalamus and SNr (E-H). D5 is present in CPu, Acb, S, hip, thalamus and SNc (I-L). Note that in Tu and SNr the distribution of D1, but not D5, is similar to Wfs1. For abbreviations, see list. Scale bar is 1 mm.

**Fig 4:**
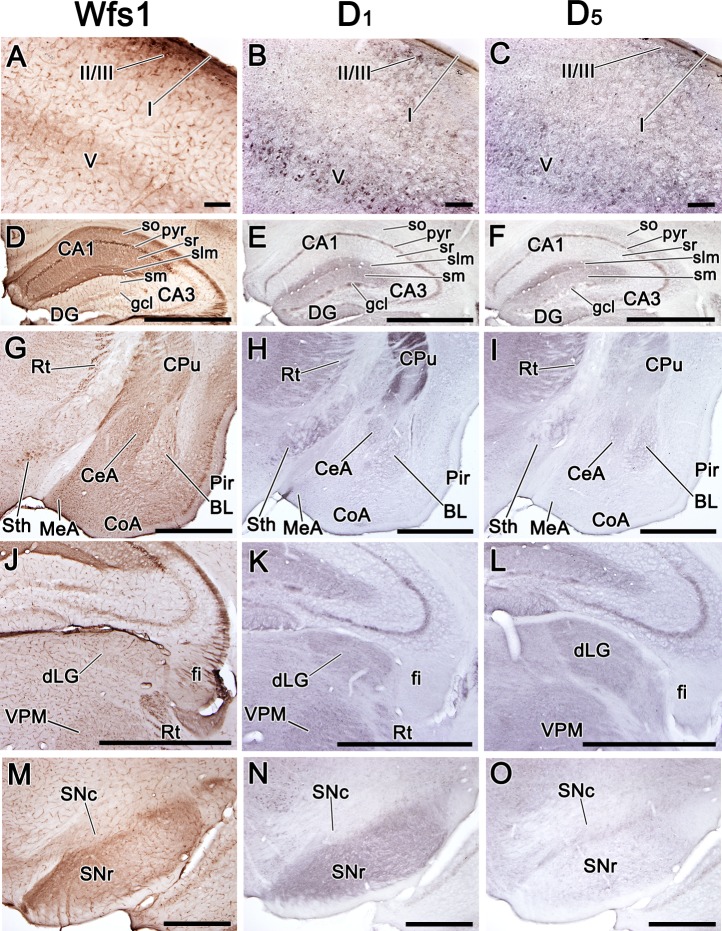
Distribution of Wfs1, D_1_, and D_5_ proteins in selected regions of the adult mouse brain. Distribution of Wfs1, D1, and D5 proteins in selected regions of the adult mouse brain. Immunohistochemistry on coronal sections. The detected proteins are indicated above. In the cortex Wfs1, D1, and D5 are all present in layer I, upper part of layer II/III, and in layer V (images show somatosensory cortex; A-C); in the hippocampus Wfs1, D1, and D5 are simultaneously present in pyr and slm of CA1 (D-F); Wfs1, D1 and D5 are all present in Rt and Sth; in amygdala Wfs1 is strongly present in CeA, the lateral edge of BL, and medial and cortical nuclei (G-I), whereas D1 and D5 show only weak signal in CeA and BL (G-I); in the dorsolateral thalamus Wfs1, D1, and D5 are delineating dLG and VPM, note that strongly Wfs1-positive fibers are present in fi, but no D1 or D5 is seen there (J-L), in the substantia nigra Wfs1 has similar distribution with D1, but not with D5 (M-O). For abbreviations, see list. Scale bar is 100 μm in A-C, 1 mm in D-L, 500 μm in M-O.

Since proteins in neurons can be transported beyond long distances from their places of synthesis, we also studied the protein distribution Wfs1 and D1-like dopamine receptors. In contrast to mRNA distribution, the regional localization of Wfs1 protein was rather similar to those of the dopamine receptors, especially with D_1_ (Fig 3A–3L). In CPu, Wfs1, D_1_ and D_5_ were all extensively present (Fig 3A–3C, 3E–3G, 3I–3K). In ventral striatum, the localization of Wfs1 was highly similar to D_1_, both were present in Acb and Tu, whereas D_5_ was missing in Tu (Fig 3A, 3B, 3E, 3F, 3I and 3J). In globus pallidus (GP), low levels of D_1_ and D_5_ were present ubiquitously, but Wfs1 was only present in the caudal part of the external segment of GP (Fig 3B, 3C, 3F, 3G, 3J and 3K). In the isocortex, Wfs1 co-occurred with D_1_ and D_5_, all were present in layer I and in the uppermost part of layer II/III, as well as in layer V (Fig 3A–3D; Fig 4A, 4B and 4C). In layer I and II/III, Wfs1 localized to both cell bodies and neuropil, but in layer V, it only appeared to be expressed in neuropil, whereas D_1_ and D_5_ receptors were present in both cell bodies and neuropil in layers II/III and V (Fig 4A, 4B and 4C). However, in the lateral cortical areas, where Wfs1 was present at high levels, the levels of D_1_ and D_5_ receptors were very low (Fig 3A–3D, 3E–3H and 3I–3L). In the hippocampus, Wfs1 was present in all layers of the CA1 region, but D_1_ and D_5_ receptors were present in the pyramidal layer and in the stratum lacunosum-moleculare of the whole CA region as well as in the stratum moleculare and weakly present in the granular cell layer of the DG (Fig 4D, 4E and 4F). Thereby, in the pyramidal layer and in the stratum lacunosum-moleculare, Wfs1 and D1-like dopamine receptors were present simultaneously. In the amygdala, Wfs1 was more widely distributed than D_1_ or D_5_, occupying CeA, BL, basomedial nucleus (BM), medial nucleus (MeA) and cortical amygdala (CoA), whereas notable amounts of D_1_ and D_5_ were only present in CeA and BL (Fig 3C, 3D, 3G, 3H, 3K and 3L; Fig 4G, 4H and 4I). Wfs1 and D_1_, but not D_5_, were strongly present in IA (Fig 2C and 2D).

In addition, we observed overlapping distribution domains of Wfs1 and D_1_-like dopamine receptor proteins in the diencephalon, where they delineated the ventral posteromedial nucleus (VPM), ventral posterolateral nucleus (VPL), reticular nucleus (Rt), dorsal lateral geniculate nucleus (dLG) and paraventricular nucleus (PV) of the thalamus (Fig 3C, 3G and 3K; Fig 4G, 4H, 4I, 4J, 4K and 4L). In the medially extended region of the subthalamic nucleus (Sth), Wfs1 was strongly present and showed overlapping localization with D_1_ (Fig 4G and 4H). In midbrain, Wfs1 was abundant in the SNr, as was D_1_ (Fig 3D and 3H; Fig 4M and 4N). In SNc and VTA, Wfs1 was present at lower levels compared to SNr, but still occupied the same domains as D_1_ (in VTA) and D_5_ (in VTA and SNc; Fig 3D, 3H and 3L; Fig 4M, 4N and 4O).

### The expression of *Wfs1* and *Drd1a* in the avian brain

#### Wfs1

**Fig 5:**
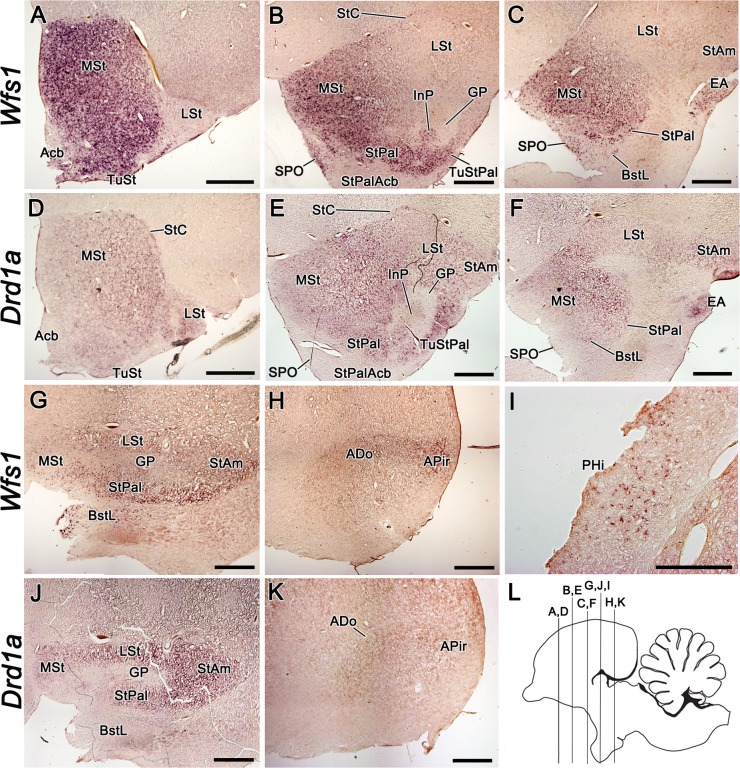
The expression of *Wfs1* and *Drd1a* in the adult chick brain. The expression of *Wfs1* and *Drd1a* in the adult chick brain, shown by mRNA *in situ* hybridization on coronal brain sections. The section plane is shown on image L. The probes are indicated on the left side of the figure. Both, *Wfs1* and *Drd1a* show strong expression in rostral to medial MSt (A-F). In Acb and StPalAcb, the expression of *Wfs1* is substantially weaker than in the surrounding striatal structures (A-B). The expression of *Drd1a* is weak in Acb and StPalAcb, and is missing in SPO (D-F). In LSt, both *Wfs1* and *Drd1a* expression show strengthening gradient in rostrocaudal direction (A-G,J). *Wfs1*-expressing cells in PHi are shown in higher magnification (I). In the adult brain, ADo and APir are delineated with *Wfs1* expression, but remain hardly distinguishable by *Drd1a* expression (H,K). Note that GP is devoid of both *Drd1a* and *Wfs1* (B,E,G,J). For abbreviations, see list. Scale bar is 1 mm in A-H and J-K and 500 μm in I.

We aimed to study the expression of *Wfs1* in parallel with *Drd1a* in the adult and developing chick brain. The results from the developmental studies are detailed in S1 Text, since the developmental expression of both genes was rather similar to adult pattern. Throughout chick brain development, the strongest *Wfs1* expression was observed in the rostral part of the medial striatum (MSt; Fig 5A and 5B; S1A and S1F Fig; S2A Fig). In the lateral striatum (LSt), *Wfs1* expression was considerably weaker in all ages and in contrary to MSt, possessed a strengthening gradient in the rostrocaudal direction (Fig 5B, 5C and 5G; S1B and S1F Fig; S2A and S2B Fig). Continuous to the MSt, the striopallidal area (StPal) showed relatively strong expression of *Wfs1* in all studied ages (Fig 5B, 5C and 5G; S1A, S1F and S1G Fig; S2A and S2B Fig). In the central component of the StPal, the intrapeduncular nucleus (InP), weak *Wfs1* expression was observed throughout the development only in the rostral part (Fig 5B; S2A and S2B Fig). In the striatal and striopallidal part of the olfactory tubercle (TuSt and TuStPal, respectively), *Wfs1* expression was low to moderate during the development, but gained strength by adulthood (Fig 5A and 5B; S1A Fig). In nucleus accumbens, *Wfs1* signal was present only in the rostral part in the adult brain and lacking in the more caudal striopallidal area of the accumbens nucleus (StPalAcb) and in the developing brain (Fig 5A and 5B; S1A and S1F Fig). The globus pallidus and ventral pallidum were devoid of *Wfs1* mRNA (Fig 5B and 5G; S1B, S1F and S1G Fig). Beginning from E15, *Wfs1* expression was also present in the striatal capsule (StC), a thin structure surrounding the striatum at the interface with the pallio-subpallial border, first described by Puelles et al., 2007 (Fig 2E; Fig 5B; S1A and S1G Fig). In the amygdala, *Wfs1* was expressed in all subpallial and some pallial regions. In the lateral part of the bed nucleus of stria terminalis (BstL), which is a subdivision of the pallidal part of the central extended amygdala [33], *Wfs1* signal was present already at E13 and remained there throughout development, although the expression domain became considerably weaker and narrower by adulthood (Fig 5C and 5G; S1B and S1G Fig; S2A and S2B Fig). Adjacent to the BstL, in the striopallidal organ (SPO), the expression of *Wfs1* was relatively strong at all developmental stages from E15 (Fig 5B and 5C; S1A, S1B, S1F and S1G Fig). In the medial septal nucleus, weak *Wfs1* expression was present only in the adult brain (S3A and S3C Fig). In the strioamygdaloid transition area (StAm) and extended amygdala (EA), amygdalar divisions of striatal origin, *Wfs1* expression was present at E13 (S2A and S2B Fig). In the rostral part of StAm the signal appeared to fade by adulthood, but persisted at moderate level in more caudal sections (Fig 5C and 5G; S1B and S1G Fig). In EA, the signal became stronger by adulthood (Fig 5C; S1B and S1G Fig). In the pallial amygdalar regions, *Wfs1* expression was present in the dorsal region of amygdala (ADo), amygdalopiriform transition area (APir), amygdaloid taenial nucleus (ATn), parataenial area of the amygdala (APTn) and the core nucleus of the amygdala, part 4 (ACo4), in late embryonic stages, peaking at E20 (S1H Fig; S2C and S2D Fig). By adulthood, the signal had faded in these structures, being faintly present only in ADo and APir (Fig 5H). Another pallial region expressing *Wfs1* was the parahippocampal area (PHi), where a few distinct cells were *Wfs1* positive in the adult brain (Fig 5I). We did not observe *Wfs1* expression in the diencephalon and midbrain of the chick.

**Fig 6:**
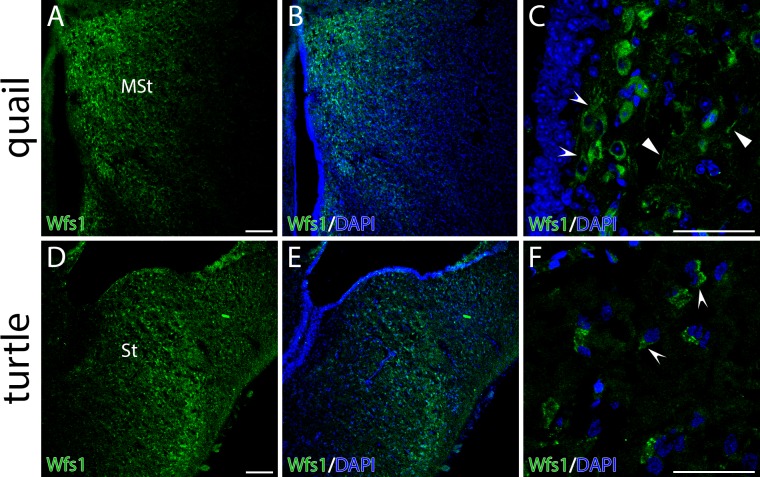
Distribution of Wfs1 protein in the striata of common quail (*Coturnix coturnix*) and red-eared slider turtle (*Trachemys scripta*) brains. Distribution of Wfs1 protein in the striata of quail (*Coturnix coturnix*) and red-eared slider turtle (*Trachemys scripta*) brains. The panel shows fluorescent immunohistochemistry on coronal brain sections. Wfs1 expression (green) is seen in medial striatum of quail (A, MSt) and turtle (D, St). Concave arrowheads point the expression in soma in both species (C, F). Wfs1 is detectable in neuronal processes of quail (C, concave arrowheads). Nuclei are counterstained with DAPI (blue). Scale bar is 100 μm.

To investigate the distribution of Wfs1 protein in avian brain, the species closely related to chick, the common quail, was used. The quail brain is smaller compared to chick brain and therefore easier to handle. At the protein level the anatomical localization of Wfs1 in MSt was similar to its mRNA expression (Fig 6A and 6B). At the cellular level Wfs1 was detectable in neuronal somas as well as in neural processes in MSt (Fig 6C) as it has been previously shown in mouse [20].

#### Drd1a

The expression domains of *Wfs1* and *Drd1a* greatly overlapped in the developing and adult brain with only few minor exceptions. Therefore, instead of describing the spatiotemporal expression of *Drd1a* in detail, we hereby point out the major differences from *Wfs1* expression pattern. The developmental dynamics and expression domains of *Drd1a* were similar to *Wfs1* in the chick striatum. There was considerably stronger *Drd1a* signal in LSt and in the lateral part of the developing rostral MSt compared to *Wfs1* signal (Fig 5A–5F, 5G and 5J; S1A–S1G, S1I and S1J Fig). A strong signal for *Drd1a* was seen in the Acb of newly hatched chick, which faded by adulthood (Fig 5D; S1I Fig), whereas there was no *Wfs1* expression in the Acb in the developing brain (S1A and S1F Fig). Another structure showing transient *Drd1a* expression was the pallidoseptal transition area (PalSe; S1J Fig). Adjacent to PalSe, the medial septal nucleus was expressing *Drd1a*, but not *Wfs1*, during E20 –P5 (S3B and S3D Fig). Contrarily, there were two subpallial regions where *Wfs1* expression was prevailing over *Drd1a*: in BstL, *Drd1a* signal was present during the development, but the expression domain diminished by adulthood (Fig 5F and 5J; S1E and S1J Fig), and in SPO, no *Drd1a* expression was detected in any stage (Fig 5E amd 5F; S1D, S1E and S1J Fig). In ADo and APir, where *Wfs1* expression was downregulated to low levels by adulthood, *Drd1a* signal faded to almost the limit of detectability (Fig 5K; S1K Fig). Transient *Drd1a* expression was in the embryonic brain in several pallial regions including the visual nidopallial nucleus, nidopallial island field, the superficial region of the intermediate nidopallium, caudolateral nidopallium and ventral mesopallium; these regions did not express *Wfs1* (S2E and S2F Fig). There was no *Drd1a* expression in PHi, where *Wfs1* was expressed in adult chick.

### The expression of *Wfs1* and *Drd1a* in the turtle brain

**Fig 7:**
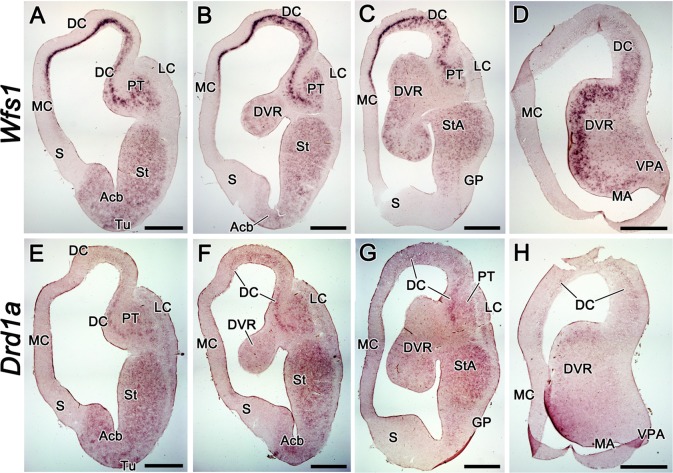
The expression of *Wfs1* and *Drd1a* in the adult red-eared slider turtle (*Trachemys scripta*) brain. The expression of *Wfs1* and *Drd1a* in the adult red-eared slider turtle (*Trachemys scripta*) brain, shown by mRNA *in situ* hybridization on coronal brain sections. The sections are in anterio-posterior order from left to right. The probes are indicated on the left side of the figure. *Wfs1* expression is widespread in the brain of *T*.*scripta*, being distinguishedly strong in MC, DC, PT and near the ventricular surface in the caudal DVR (A-D). *Drd1a* expression occupies the same regions as that of *Wfs1*, but is missing in MC and very weak in DC and caudal DVR (E-H). Unlike *Wfs1*, *Drd1a* is present in LC (E-G). For abbreviations, see list. Scale bar is 1 mm.

We detected *Wfs1* and *Drd1a* expression in adult *T*. *scripta* brain using RNA probes specific to chick mRNA. As in the chick, we observed significant overlap of *Wfs1* and *Drd1a* expressions in the turtle forebrain. Both genes had widespread expression, showing mRNA signal in subpallium as well as in numerous pallial regions. In subpallium, both were expressed in striatal and amygdalar territories including striatum (St), Acb, striatoamygdalar area (StA) and medial amygdala (MA), whereas GP and septum were devoid of expression (Fig 7A, 7B, 7C, 7E, 7F and 7G). In pallial structures, the expression patterns of these two genes were similar but not completely identical. *Wfs1* showed prominent expression continuously in mammalian hippocampal homologue medial cortex (MC), isocortical homologue dorsal cortex (DC) and in the pallial thickening (PT), a lateral pallial derivative supposedly homologous to the claustrum/endopiriform formation [34] (Fig 7A, 7B, 7C and 7D). We could not detect *Drd1a* expression in MC and observed only weak signal in DC and moderate signal in PT (Fig 7E, 7F, 7G and 7H). Conversely, there was weak expression of *Drd1a* but not of *Wfs1* in lateral cortex (LC; Fig 7A, 7B, 7C, 7E, 7F and 7G). In the dorsal ventricular ridge (DVR), *Wfs1* was expressed relatively strongly in cell clusters near to the ventricular side, especially in the caudal part (Fig 7B, 7C and 7D). With the *Drd1a* probe, the expression pattern was similar but equally weak in rostral and caudal DVR (Fig 7F, 7G and 7H). In the ventral posterior amygdala (VPA), a pallial region proposed to be homologous to the posterior division of MeA or amygdalo-hippocampal transition area in the mammalian brain [35–36], weak *Wfs1* expression, but no *Drd1a* expression, was present. Like in chick, but unlike the mouse, we could not detect *Wfs1* expression in the diencephalon and midbrain of *T*. *scripta*.

At the protein level, we show that the anatomical localization of Wfs1 recapitulates Wfs1 mRNA (Fig 6D and 6E). At the cellular level, turtle Wfs1 localizes predominantly in the soma of the neurons (endoplasmatic reticulum and axon hillock; Fig 6F).

**Fig 8:**
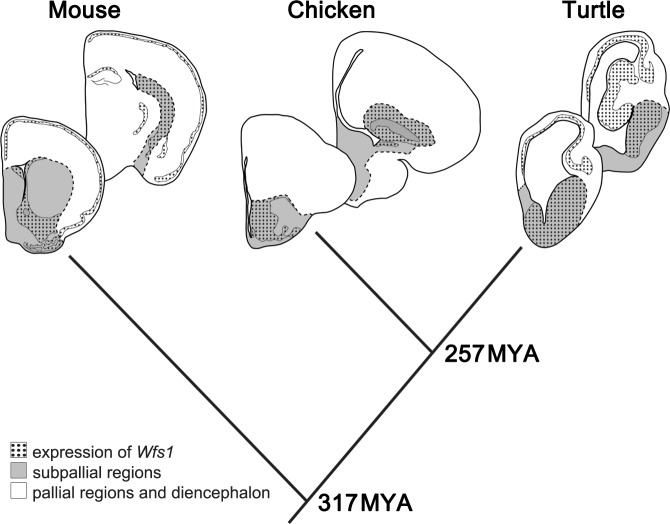
The expression of *Wfs1* in pallial and subpallial regions of mouse, chick and red-eared slider turtle brain. Schematic depiction of the expression of *Wfs1* (dotted area) in pallial (white) and subpallial (grey) regions of mouse, chick and red-eared slider turtle brain. Coronal sections. Times of evolutionary divergence are based on [37]. Dashed line—border delineating subpallial regions.

Overall, the expressions of *Drd1a* and *Wfs1* significantly overlapped in several regions of the brains of the three studied species. Especially in chick and turtle brain, the distribution of *Wfs1* mRNA almost completely paralleled the expression pattern of *Drd1a*. Consistent with the extent of evolutionary conservation of subpallial versus pallial structures, the expression of both genes was more conserved in subpallial structures compared to pallial regions of the studied species. The expression of *Wfs1* in pallial *versus* subpallial structures in the mouse, chick and red-eared slider turtle brain is illustrated in Fig 8. A summary of *Wfs1* and *Drd1a* expression in the brain structures of the studied species is shown in Table 1 and a detailed discussion on the main findings is in S2 Text.

**Table 1:** *Wfs1* and *Drd1a* presence and relative expression in homologous brain structures of mouse, chick and red-eared slider turtle.

Region (mammalian/avian/reptilian)	Mouse protein	Mouse mRNA	Chick mRNA	Turtle mRNA	homology proposed by
**Pallial regions**					
neocortex/hyperpallium/DC	• Wfs1+++ • D_1_+	• *Wfs1*+++ • *Drd1a*+	-	• *Wfs1*+++ • *Drd1a*+	[34]
hip/hip/MC	• Wfs1+++ • D_1_+++	• *Wfs1*+++ • *Drd1a*+	-	• *Wfs1*++ • *Drd1a*-	[34]
pir /pir /LC	• Wfs1+++ • D_1_+	• *Wfs1*+++ • *Drd1a*+	-	• *Wfs1+++ • *Drd1a*-	[34]
Claustrum and endopiriform/mesopallium/PT	• Wfs1- D_1_+	• *Wfs1*- • *Drd1a*+	• *Wfs1- • *Drd1a+	• *Wfs1*+++ • *Drd1a++	[34]
lateral amygdala/sensory nidopallium/anterior DVR	• Wfs1+ • D_1_+	• *Wfs1*+ • *Drd1a*+	• *Wfs1*- • *Drd1a*+	• *Wfs1*+ • *Drd1a+	[34]
BL/ADo/caudal DVR	• Wfs1++ • D_1_+	*Wfs1*++ • *Drd1a*+	• *Wfs1*+ • *Drd1a*+	• *Wfs1*+++ • *Drd1a+	[34]
BM/ACo/caudal DVR	• Wfs1+++ • D_1_+	• *Wfs1*++ • *Drd1a*+	• *Wfs1+ • *Drd1a*-	• *Wfs1*+++ • *Drd1a*+	[34]
CoA (posterolateral) and APir/APir/LC	• Wfs1+++ • D_1_+	• *Wfs1*++ • *Drd1a*+	• *Wfs1*+ • *Drd1a*+	• *Wfs1*- • *Drd1a*+	[36]
AHi/AHi/VPA	• Wfs1+++ • D_1_+	• *Wfs1*+ • *Drd1a*+	• *Wfs1*+ • *Drd1a*-	• *Wfs1*+ • *Drd1a*-	[36]
**Subpallial regions**					
anterior. . .posterior CPu/MSt/St	• Wfs1+. . .+++ • D_1_+++	• *Wfs1-…*+++ • *Drd1a*+++	• *Wfs1*+++ • *Drd1a*+++	• *Wfs1*++ • *Drd1a*++	
anterior. . .posterior CPu/LSt/St	• Wfs1+…+++ • D_1_+++	• *Wfs1-…*+++ • *Drd1a*+++	• *Wfs1*+ • *Drd1a*++	• *Wfs1*++ • *Drd1a*++	
Acb/Acb/Acb	• Wfs1+++ • D_1_+++	• *Wfs1*+++ • *Drd1a*+++	• *Wfs1*+ • *Drd1a*+	• *Wfs1*++ • *Drd1a*++	
Tu/TuSt and TuStPal/Tu	• Wfs1+++ • D_1_+++	• *Wfs1*+++ • *Drd1a*+++	• *Wfs1*+++ • *Drd1a*+++	• *Wfs1*++ • *Drd1a*++	
GP/GP/GP	• Wfs1+++ • D_1_+	-	-	-	
ventral pallidum/PalV/not described in turtle	• Wfs1++ • D_1_+	-	-		
S/S/S	• Wfs1++ D_1_-	• **Wfs1+++ • *Drd1a*+	• *Wfs1*+ • *Drd1a++		
IA /StC/not described in turtle	• Wfs1+++ • D_1_+++	• *Wfs1*+++ • *Drd1a*+++	• *Wfs1*++ • *Drd1a*++		[38]
CeA /StAm and EA/StA	• Wfs1+++ • D_1_++	• *Wfs1*+++ • *Drd1a*+	• *Wfs1*+++ • *Drd1a*+++	• *Wfs1*++ • *Drd1a*++	[35], [38]
MeA /ATn/MA	• Wfs1+++ • D_1_+	• *Wfs1*++ • *Drd1a*+	• *Wfs1+ • *Dd1a*-	• *Wfs1*++ • *Drd1a*+	[35], [38]
BstL/BstL	• Wfs1++ • D_1_+	• *Wfs1*++ • *Drd1a*+	• *Wfs1*++ • *Drd1a*+	nd	[36]
**Diencephalon**					
Thalamus/Thalamus/Thalamus	• Wfs1+ • D_1_++	• *Wfs1*+ • *Drd1a*+	-	-	
hy/hy/hy	• Wfs1++ • D_1_+	• *Wfs1*+ • *Drd1a*+	-	-	
**Midbrain**					
SNr/SNr/SNr	• Wfs1+++ • D_1_+++	-	-	-	
SNc/SNc/SNc	• Wfs1+ D_1_-	• *Wfs1-* • *Drd1a*+	-	-	
VTA/VTA/VTA	• Wfs1+ • D_1_+	• *Wfs1*+ • *Drd1a*+	-	-	

Scores indicate relative expression levels:

+++, high expression;

++, moderate expression;

+, low expression; -, no expression; nd, not determined;

* present only during development;

** present only in LSD. The expression assessments with particular probe are comparable within the species only.

### D1-like dopamine receptor binding is increased in *Wfs1*^-/-^ mouse hippocampi

**Fig 9:**
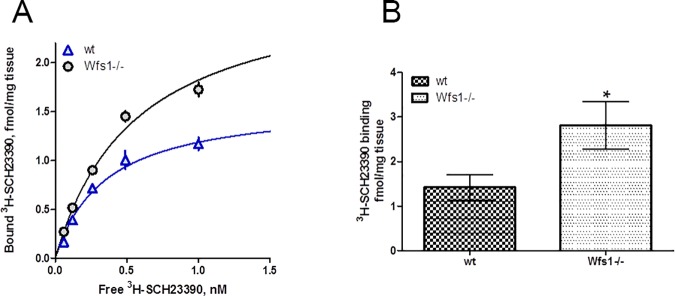
Binding of D_1_/D_5_ specific ligand [^3^H]SCH23390 to hippocampal membranes of wt and *Wfs1* knockout mice. Comparison of specific binding of radioligand [^3^H]SCH23390 to hippocampal membranes of wt and *Wfs1* knockout mice. (A) Binding curve of [^3^H]SCH23390 binding to pooled samples of wt (triangle) and *Wfs1* knockout (circle) mice. The membrane suspensions (3 mg/well) were incubated with different concentrations of [^3^H]SCH23390 for 60 min and bound radioactivity was measured. Data are presented as mean ± SEM from experiments (n = 3) performed in duplicates. (B) The level of [^3^H]SCH23390 binding sites of individual wt and *Wfs1* knockout mice determined in hippocampal membrane suspensions (6.7 mg/ml.) incubated with 4 nM radioligand. Data presented as mean ± SEM of all the mice tested. *P < 0.05. Data of individual mice are presented in S1 Table.

To determine whether Wfs1 is involved in the proper functioning of D1-like dopamine receptors, the number of binding sites of dopamine receptors in the mouse hippocampus were assayed by [^3^H]SCH23390, a specific ligand for D1-like receptors. As this radioligand does not distinguish between the two subclasses of D1-like dopamine receptors, D_1_ and D_5_, which both are expressed in hippocampus and have quite similar roles [39], the following conclusions are valid for D1-like receptors. The [^3^H]SCH23390 bound to hippocampal membranes with high affinity, having K_D_ values 0.31 ± 0.06 nM and 0.48 ± 0.08 nM (n = 3) for wt and *Wfs1* gene knockout mice, respectively (Fig 9A). The number of [^3^H]SCH23390 binding sites of *Wfs1* knockout mice (B_max_ = 4.03 ± 1.31 fmol/mg tissue) was higher than that of wt mice (B_max_ = 1.45 ± 0.10 fmol/mg tissue; Fig 9A).

To check how the number of D_1_/_5_-specific binding sites is distributed between individual mice, [^3^H]SCH23390 binding was performed at 4 nM concentration of the radioligand. At this concentration approximately 90% of available receptors are bound, giving representative information about the number of total binding sites. The value obtained for *Wfs1* knockout mice, 2.8 ± 0.5 fmol/mg tissue (n = 24), was significantly higher (p < 0.05) than corresponding value, 1.4 ± 0.3 fmol/mg tissue (n = 22), for wt mice (Fig 9B, S1 Table).

## Discussion

### Wfs1 is expressed in dopaminoceptive regions of the amniote brain and regulates dopamine signalling through D1-like receptors

We have previously shown that murine *Wfs1* expression is initiated during the late embryogenesis when massive synaptogenesis takes place. The expression of *Wfs1* is specifically strong in the brain regions involved in the emotional control of behavior and the integration of sensory and motor signals [20], [22]. Many of these regions–striatum, cerebral cortex, hippocampus and central extended amygdala–are known to be the targets of dopaminergic pathways [40–41]. Importantly, previous studies support a relationship between Wfs1 and dopamine signalling. *Wfs1*-deficient mice are less sensitive to locomotor stimulatory effect of amphetamine and more sensitive to that of apomorphine, compared to wild-type mice, suggesting both pre- and postsynaptic changes in dopaminergic synapses [24–25]. *Wfs1* deficient mice also have lower ability to secrete dopamine in the striatum [23].

Studying the possible relations between Wfs1 and dopamine receptors is therefore crucial for understanding the etiology and pathophysiology of the psychiatric symptoms of Wolfram syndrome patients carrying mutant alleles at this locus [15], [18], [42–43].

Wfs1 has been shown to regulate positively the synthesis of cyclic AMP in pancreas [11]. Therefore, we focussed specifically on the involvement of Wfs1 in D1-like dopamine receptor signalling, which in contrast to D2-like receptor signalling, is positively coupled to adenylyl cyclase activity [26]. We found that the localization of Wfs1 and D1-like dopamine receptors coincide at the protein level in several regions of the mouse brain. Furthermore, in evolutionarily distant species, in the chick and turtle brain, *Wfs1* and *Drd1a* exhibited remarkable overlap in their expression regions, suggesting further for the co-operativity of these proteins. To shed more light into this subject we performed a D1-like dopamine receptor specific radioligand binding assay in the hippocampi of *Wfs1*^-/-^ and wt mice. *Wfs1* deficiency resulted in the increase of the D1-like dopamine receptor binding sites, confirming that the postsynaptic dopamine signalling is altered. The upregulation of D1-specific binding might be a compensatory change in order to maintain sufficient levels of dopaminergic signalling in case of reduced dopamine output from the midbrain. Additionally, increase in the number of D1-like receptors may occur due to possible abnormal signal transduction from D1-like receptors in *Wfs1*^*-/-*^ mice. One proposed role of Wfs1 is to regulate endoplasmic reticulum (ER) stress induced unfolded protein response [4], [5], [9]. Dimerization of several G-protein coupled receptors that function as homo- or heterodimers occurs in ER. Likewise, balanced ER function is needed for D1-like dopamine receptor dimerization that form both homodimers and heterodimers with adenosine A1 receptor [44]. ER stress caused by Wfs1 deficiency could therefore lead to improper receptor biogenesis, which also might lie behind the alterations in the expression of D1-like receptors. To address these questions, further studies are needed to measure the activity of the intracellular signalling pathways of D1-like receptors and receptor folding/biogenesis in *Wfs1* deficient mice.

The dopaminergic fibers in the forebrain originate from the dopaminergic cells located in the midbrain in the substantia nigra and ventral tegmental area. The substantia nigra is divided into dopaminergic pars compacta (SNc) and GABA-ergic pars reticulata (SNr). The activity of the dopaminergic cells in the SNc is largely under control of the GABA-ergic cells of SNr, which, in turn, receive GABA-ergic inhibition via striatonigral and pallidonigral afferents [45–46]. Striatonigral afferents reaching the SNr contain D_1_ receptors [47], which, upon activation, promote the GABA-ergic inhibition of SNr cells and thus decrease the inhibitory input to SNc [48–49]. A strong Wfs1 immunoreactive striatonigral projection probably arising from the Acb core has been described in mouse SNr [20]. The ramification of this projection is seen in the SNr in Fig 3M and 3N, and it shows immunoreactivity to both, Wfs1 and D_1_. If D_1_ signalling in the striatonigral afferents innervating SNr is affected by the loss of Wfs1, the GABAergic control over SNc should also be affected and SNc dopamine output altered. Our data show for the first time the link between Wfs1 and D1-like dopamine signalling, however more knowledge is needed to understand the entire physiological importance of this link.

The use of *Wfs1* and *Drd1a* expression pattern to confirm or refute hypotheses of homologous brain region function between vertebrate groups is discussed in S2 Text. In most instances, the mouse, chick, and turtle have similar expression patterns, allowing similar functions to be ascribed to particular regions. Moreover, specific differences in the expression of *Wfs1* between vertebrate brains may have important functional significance. In mammals, for instance, the hippocampus, and especially its CA1 region, which strongly expresses *Wfs1*, has been shown to be very susceptible to neuronal death caused by cerebral ischaemia and the related glutamatergic excitotoxicity [50–51]. Interestingly, the brains of freshwater turtles are known to be highly resistant to hypoxic/ischaemic and glutamate-related neuronal damage [52–53]. Several gene and protein expression patterns can be attributed to reflect the ability of turtle neurons to survive hypoxia [54–60]. Arising from this argumentation, it is intriguing to hypothesize that the remarkably strong expression of *Wfs1* seen in the medial and dorsal cortices of *T*. *scripta* is related to the resistance of hypoxia in these animals. In many cases of ischaemic and excitotoxic brain damage, activation of calpains, a family of calcium-dependent proteases, leads to apoptosis via cleavage of caspases [61–62]. The increased calpain activity occurring in *Wfs1* deficiency [63] might link it to the resistance to hypoxia.

## Concluding remarks

*Wfs1* is a gene encoding Wolframin, a protein involved in mitigating ER stress, regulating insulin secretion from pancreatic β-cells, coordinating cellular Ca^2+^ homeostasis, and stabilizing the folding of several proteins. In the mammalian brain, it is expressed in several regions associated with emotional control of behavior. Our immunohistochemical study in mouse brain showed that the distribution of Wfs1 was largely overlapping with that of D1-like dopamine receptors, especially with D_1_. Previously, alterations in the functioning of the dopaminergic system have been shown in mice deficient for *Wfs1* gene. We present here the first evidence for the interaction of Wfs1 and the dopaminergic receptor pathway to give relevance to the anatomical localizations that we found. Our study suggests that alterations in dopaminergic signalling are caused, at least in part, by the upregulation of D1-like dopamine receptor density in *Wfs1*^-/-^ mice. The dysregulation in dopaminergic system might be the underlying cause of the psychiatric findings in Wolfram syndrome patients and carriers of mutant allele. In order to better understand the evolutionary context of the relation between Wfs1 and D1-like dopamine receptors, we performed an *in situ* hybridization study of *Wfs1* and *Drd1a* genes in the brains of domestic chick and red-eared slider turtle, representatives of birds and chelonian reptiles, respectively. The conservation of the coexpression of *Wfs1* and *Drd1a* in many brain regions of the studied animals underscores the important link between the two genes. Orchestrating the behavioral responses to environmental stimuli, the interaction between Wfs1 and D1-like dopamine receptors is an intriguing substrate for evolutionary adaptations.

## Supporting information

S1 Text Chick development studies. (DOCX)

S2 Text Comparisons of Wfs1 and Drd1a expression in the brain between three amniote lineages. (DOCX)

S1 Fig The expression of *Wfs1* and *Drd1a* in the developing chick brain, shown by mRNA *in situ* hybridization on coronal brain sections. Medial side of the sections is on the left and lateral side on the right. The section plane is shown in image C. The probes are indicated on the left side of the figure and stages are indicated in the images. Note that in the lateral part of MSt and in anterior LSt, *Drd1a* is present, but not *Wfs1* (compare A to D and F to I). In SPO, *Wfs1* is expressed, but not *Drd1a* (compare A to D, B to E, F to I, G to J). In Acb, *Drd1a* is expressed, but not *Wfs1* (compare A to D, F to I). For abbreviations, see list. Scale bar is 1mm.
(TIF)

S2 Fig The expression of *Wfs1* and *Drd1a* in selected regions of the developing chick brain, shown by mRNA *in situ* hybridization on coronal brain sections. Medial side of the sections is on the left and lateral side on the right. The probes are indicated on the left and stages are indicated on the images. By E13, most of the subpallial regions were expressing *Wfs1* (A,B). In pallial amygdala, the expression of *Wfs1* was most widespread at E20 (C,D). Several regions of the nidopallium were expressing *Drd1a* in developing brain (E,F). For abbreviations, see list. Scale bar is 1mm.
(TIF)

S3 Fig The expression of *Wfs1* and *Drd1a* in the medial septal nucleus in developing (E20) and adult chick brain, shown by RNA *in situ* hybridization on coronal brain sections. Medial side of the sections is on the left and the lateral side on the right. The probes are indicated on the top and stages are indicated on the left. Note that during the development, only *Drd1a* is present in MS (compare A and B), but in adulthood, only *Wfs1* is present in the same structure (compare C and D). For abbreviations, see list. Scale bar is 1mm.
(TIF)

S1 Table The number of binding sites of D1-like receptors in hippocampal membranes of wt and *Wfs1* knockout mice. The binding of 4 nM [^3^H]SCH23390 was determined in duplicates or triplicates in the absence (for total binding) or in the presence (for nonspecific binding) of 10 μM (+)-butaclamole at tissue concentration 6.7 mg/ml. The specific binding was calculated as difference between total and nonspecific bindings and presented as mean value for particular mouse.
(DOCX)

## Abbreviations

Acb: nucleus accumbens
ACo4: core nucleus of the amygdala, part 4
aCPu: anterior caudate-putamen
ADo: dorsal region of the amygdala
Am: amygdala APir, amygdalopiriform transition area
APTn: parataenial area of the amygdala
ATn: amygdaloid taenial nucleus
BL: basolateral amygdala
BM: basomedial amygdala
BST: bed nucleus of the stria terminalis
BstL: bed nucleus of the stria terminalis, lateral part
CA1: *cornu ammonis* 1 region of the hippocampus
CA3: *cornu ammonis* 1 region of the hippocampus
CeA: central nucleus of the amygdala
CoA: cortical amygdala
CPu: caudate-putamen
Cx: cerebral cortex
D_1_: dopamine receptor 1
D_2_: dopamine receptor 2
D_5_: dopamine receptor 5
DAB: diaminobenzidine
DC: dorsal cortex
DG: dentate gyrus
Dig: digoxygenin
dLG: dorsal lateral geniculate nucleus of the thalamus
DTT: dithiothreitol
DVR: dorsal ventricular ridge
E: embryonic day
EA: extended amygdala
ER: endoplasmatic reticulum
fi: fimbria
gcl: granule cell layer of the dentate gyrus
GP: globus pallidus
HB: homogenization buffer
hip: hippocampus
hy: hypothalamus
IA: intercalated amygdala
IB: incubation buffer
InP: intrapeduncular nucleus
IPAC: interstitial nucleus of the posterior limb of he anterior commissure
LC: lateral cortex
LHb: lateral habenula
LSD: dorsal nucleus of lateral septum
LSt: lateral striatum
MA: medial amygdala
MC: medial cortex
MeA: medial nucleus of the amygdala
MS: medial septal nucleus
MSt: medial striatum
NCS: nidopallium, caudal part, superficial region
NIF: nidopallial island field
NIS: nidopallium, intermediate part, superficial region
PalSe: pallidoseptal transition area
PBS: phospate buffered saline
pCPu: posterior caudate-putamen
PFA: paraformaldehyde
PHi: parahippocampal area
Pir: piriform cortex
PT: pallial thickening
PV: paraventricular nucleus of the thalamus
pyr: pyramidal layer of the *cornu ammonis*
Rt: reticular nucleus of the thalamus
S: septum
SEM: standard error of mean
slm: *stratum lacunosum-moleculare* of the *cornu ammonis*
sm: *stratum moleculare* of the dentate gyrus
SNc: *substantia nigra*, *pars compacta*
SNr: *substantia nigra*, *pars reticulata*
so: *stratum oriens* of the *cornu ammonis*
SPO: striopallidal organ
sr: *stratum radiatum* of the *cornu ammonis*
St: striatum
StA: striatoamygdalar area
StAm: strioamygdaloid transition area
StC: striatal capsule
Sth: subthalamic nucleus
StPal: striopallidal area
StPalAcb: striopallidal area of the nucleus accumbens
Tu: olfactory tubercle
TuSt: striatal part of hte olfactory tubercle
TuStPal: striopallidal part of the olfactory tubercle
VisCo: visual nidopallial nucleus, core region
VPA: ventral posterior amygdala
VPL: ventral posterolateral nucleus of the thalamus
VPM: ventral posteromedial nucleus of the thalamus
VTA: ventral tegmental area
Wfs1: Wolfram syndrome 1
