# Supplementary material for: Wfs1 is expressed in dopaminoceptive regions of the amniote brain and modulates levels of D1-like receptors
Source: PLoS One. 2017 Mar 7;12(3):e0172825. doi: 10.1371/journal.pone.0172825 (PMC5436468; doi:10.1371/journal.pone.0172825)
Supplement: S1 Text — (DOCX) [file pone.0172825.s001.docx]

**S1 text. Chick development studies**

No *Wfs1* signal was detectable at E10, the age comparable to mouse E16, roughly the ending of the peak of neuronal migration. At E13, weak expression was present in several subpallial brain regions including MSt, LSt, StPal, InP and in subpallial domains of extended amygdala including BstL, StAm and EA (S2 Fig A,B).
