# Supplementary material for: Wfs1 is expressed in dopaminoceptive regions of the amniote brain and modulates levels of D1-like receptors
Source: PLoS One. 2017 Mar 7;12(3):e0172825. doi: 10.1371/journal.pone.0172825 (PMC5436468; doi:10.1371/journal.pone.0172825)
Supplement: S2 Text — (DOCX) [file pone.0172825.s002.docx]

**S2 text. Comparisons of *Wfs1* and *Drd1a* expression in the brain between three amniote lineages.**

After diverging about 320 million years ago, mammalian and sauropsid brains have evolved separately, elaborating the ancestral structures in different ways. More recently, about 257 million years ago, the chelonian (turtle) lineage diverged from the archosaurian lineage containing the ancestors of birds and crocodilians [1-2]. As a result of the evolutionary adaptations, the pallium, serving more specialized fine-tuned brain functions, underwent accelerated growth and elaboration compared to the subpallium in mammalian and avian lineages.

In mammals, most of the pallial part of the forebrain is comprised of a six-layered neocortex and allocortical areas including hippocampus and olfactory cortices, which contain a smaller number of layers. The amygdala, which contains both pallial and subpallial parts, is mostly built up in a nuclear manner, similarly to the subpallium.

In reptiles and birds, pallial structures have mostly nuclear or a less conspicuously stratified organization. A unique feature of sauropsid pallium is the dorsal ventricular ridge (DVR), a thickening of the lateral wall of the anterior neural tube that protrudes into the lateral ventricle. The DVR functions as the major integratory centre in the sauropsid brain analogously to the neocortex of mammals. In birds, DVR is continuous with the overlying pallial structure - the hyperpallium, but in the reptilian brain it is separated from the uppermost structure, the dorsal cortex, by the lateral ventricle [3].

Establishing the homologies for the forebrain structures between amniote lineages has been a difficult matter evoking continuous disputes [4-11]. Common developmental origin does not necessarily imply that the function of homologous structures has been retained similar during evolution (anymore than the homology of the mammal`s jaw and a fish gill arch would predict similar function). To this end, the expression of genes characteristic to particular pathways and networks for particular functions is critically important to assess [12].

It seems highly probable that Wfs1 is located in brain structures involved in common neural networks and affected by common stressors. In the brain, clear evidence exists showing the involvement of Wfs1 in proper dopamine signaling [13-14], and here we show that Wfs1 is specifically related to D1-like dopamine receptor signalling. Importantly, D1-like dopamine receptor-mediated signalling can by itself act as a stressor for the postsynaptic cell, upregulating ER stress and apoptosis pathways [15-16]. This evidence allows us to consider Wfs1 as a functional marker of brain structures and can help assess which regions of the brain may be processing similar inputs. Therefore, in addition to mammals, we compared the expression patterns of *Wfs1* and D1-like dopamine receptor *Drd1a* also in a representative of birds (domestic chick *Gallus gallus*), and chelonian reptiles (red-eared slider turtle *Trachemys scripta*).

**Striatum**

In the mouse striatum, *Wfs1* mRNA is present ubiquitously during development, but is restricted to the ventral striatum and caudal CPu in adulthood [17-18]. *Wfs1* was also expressed in the avian counterparts of the mammalian CPu [19]: strongly in MSt and weakly in LSt. Similar gradient in expression intensity was evident for *Drd1a* in the chick brain, which is also pointed out by previous authors, and hypothesized to reflect the medial to lateral gradient in the distribution of striatonigral versus striatopallidal projection neurons [20]. While the dorsal striatum/CPu is involved mostly in generating movements and associative learning, the ventral striatum, which includes the Acb and Tu in mouse, belongs to the emotional circuitry of the brain [21-22]. By preferential connections with the limbic pallial regions and VTA, as well as developmental gene expression patterns and neurochemical characteristics, the ventral striatal territories have also been recognized in the avian telencephalon [8], [19], [23-25].

*Wfs1* was widely distributed in the avian ventral striatum, including StPal, InP, TuStPal and TuSt, but, contrasting to the mouse, the Acb of the chick was largely devoid of expression. The low expression of *Wfs1* in the chick Acb paralleled the low levels of *Drd1a* mRNA. In line with our finding, previous studies have shown that the avian Acb is relatively poor in DARPP-32, a phosphoprotein mediating the intracellular signalling of D_1_ receptor binding [26-27], and shows low density of D_1_, but high density of D_2_ receptor binding sites [28-29]. This contrasts with the situation in mammals, where D_1_ and DARPP-32 are present at high levels in the Acb [30-32]. It is also worth of mention that defining the precise location and extent of the avian Acb has been a matter of controversy [19-33]. Still, Wfs1 and D_1a_ may mutually take part in some of the functions of Acb in birds, since it has been proposed that the avian Acb might extend into the mediodorsal MSt [34], which was strongly expressing both *Wfs1* and *Drd1a*.

In turtle brain, all striatal areas, including Tu, Acb and St were expressing *Wfs1*, as well as *Drd1a*, suggesting that the diminishing of *Wfs1* expression in the chick Acb probably happened after the divergence of chelonian lineage.

Interestingly, *Wfs1*, but not *Drd1a,* was expressed in the chick SPO, a circumventricular organ in the striatal division of the forebrain described in [35] and [36]. In mammalian brain, Wfs1 expression is also present in circumventricular structures, namely in the magnocellular parts of the hypothalamic supraoptic and paraventricular nuclei [37], where it is possibly involved in regulating the processing of arginine vasopressin [38].

**Amygdala**

The expression of Wfs1 has been previously shown to be present in all amygdalar nuclei of the mouse brain, showing distinguishably high levels in the central extended amygdala, including the central amygdaloid nucleus, BstL and IPAC, and in the medial nucleus during the development [17-18, 37]. The central nucleus of the amygdala is the main integrative center in the amygdaloid complex, mediating autonomic, somatic, endocrine and behavioral responses to different environmental stimuli [39]. In rodents, the role of CeA has been extensively studied in the context of fear-related learning [40-41]. In restful emotional state, the CeA is under inhibitory control from the cortex through the activation of GABA-ergic neurons forming the intercalated cell masses that border the CeA and BL [41-44]. On the other hand, the intercalated cells receive strong dopaminergic projection, which has inhibitory effect through D_1_ receptors, and thus promotes the neural activity in BL and CeA upon fearful signal from the environment [44-46]. We showed that the intercalated amygdala cells coexpress strongly both, Wfs1 and D_1_ (Fig 2), indicating that Wfs1 and D_1_ receptor may interact in regulating the activity of CeA and modulating stress-related behavior. In birds, StAm and in reptiles StA are considered homologous to the mammalian central nucleus of the amygdala [4], [23], [47-50], and were both simultaneously expressing *Wfs1* and *Drd1a*. Similarly to mouse, *Wfs1* and *Drd1a* were concurrently expressed in a proposed avian homologue to the intercalated amygdala, the StC [23], [35] (see Fig 2 A-F). Our results indicate that the function of the CeA/StAm/StA in fear-related behavior is conserved in amniotes, and StC may have similar role to intercalated nuclei of the amygdala.

The developmentally heterogenous MeA is the main target of the projections from the accessory olfactory bulb, receiving and processing the chemical information related to the presence of predators or potential sexual partners [51-52]. In rodents, cat odour causes defensive behavior and neural activation in MeA [53-54], and MeA lesions are shown to abolish the fear responses to the presence of cat odour [55]. Cat odour exposure increases the expression of *Wfs1* in the amygdala of rat, yet it is not specified in which nuclei [56]. Although the main and accessory olfactory systems have widely been thought to be poorly developed in most bird species, accumulating evidence shows that this may not be the case [57-58]. In domestic chicks, predator odour induces avoidance at posthatching day 7, but not at day 10, which may reflect the relative importance of olfactory cues at early posthatching ages [59]. Interestingly, in both mouse MeA and its proposed avian homologue, ATn, the expression of *Wfs1* is transient [18], [60], suggesting its involvement in fine-tuning the odour- or pheromone-related fear circuits.

The pallial amygdala is comprised of the basolateral amygdala, which is involved in associating and relaying visual, auditory and somatosensory input, and the basomedial and cortical nuclei, which receive input from the main and accessory olfactory bulb and are involved in reproductive, defensive and ingestive behaviors [61-62]. In most of the pallial amygdala, the expression of *Wfs1* showed the same tendency in both, mouse and chick: being strong in the developing brain and diminishing by adulthood (see [18]; this study).

The pallial amygdala is subject of controversies in terms of homologies. Martínez-García et al. in [63], propose that the region corresponding to ACo is homologous to the mammalian BM and ADo belongs to the equivalent territory of mammalian basal nucleus. It is intriguing to speculate that the developmental dynamics of *Wfs1* expression in ACo may be inherited from the common ancestor of sauropsids and mammals. On the other hand, some authors propose that ADo and ACo, alternatively named “arcopallium dorsale” and “arcopallium intermedium”, respectively, by the Avian Brain Nomenclature Forum [19], are homologous to layers V and VI of the neocortex in mammalian brain [10-11], [19], [64-65]. In the pallial amygdala of the chick, the expression of *Drd1a* was similar to *Wfs1* both in terms of distribution and downregulation by adulthood suggesting the cooperation of the two proteins during the forming of amygdala circuits.

In the pallial amygdala of turtle, high *Wfs1* expression in the posterior DVR and lower expression in anterior DVR is consistent with the proposal that the posterior DVR is the reptilan homologue of the mammalian BM and the anterior DVR being homologous to the mammalian LA [4], [63].

**Cerebral cortical and hippocampal homologues**

The high evolutionary conservation of *Wfs1* expression observed in subpallial structures was not seen in the pallial regions. In mouse, *Wfs1* is expressed in the layer II/III of the neocortex, in entorhinal and piriform cortices and in the CA1 region of the hippocampus [17-18, 37]. At the protein level, overlapping localization of Wfs1, D_1_ and D_5_ was detectable in layer I, in the upper part of layer II/III and in layer V, which suggests that Wfs1 and D1-like receptors are involved in the same circuits and may functionally interact in the cerebral cortex. *Wfs1* was not expressed in the putative avian cerebral cortical homologues: hyperpallium, mesopallium and nidopallium. Also in the chick hippocampal complex, only few cells were found to be positive for *Wfs1* signal in the PHi, a region homologous to mammalian subiculum and entorhinal cortex [66]. Interestingly, the pattern of *Wfs1* expression was more similar between mouse and turtle than between turtle and chick, probably reflecting adaptive necessity rather than evolutionary homology in the respective phylogenetic clades. Namely, in turtle brain *Wfs1* was strongly expressed in the dorsal cortex and in the pallial thickening, the homologous regions of the dorsal neocortex and claustrum/endopiriform formation, respectively [60]. Mostly, *Drd1a* was seen consistently mirroring the expression pattern of *Wfs1* in the cerebral cortical homologues of both chick and turtle.


**References**

1. Crawford NG, Faircloth BC, McCormack JE, Brumfield RT, Winker K, Glenn TC. More than 1000 ultraconserved elements provide evidence that turtles are the sister group of archosaurs. Biol Lett. 2012;8(5): 783-786.

2. Wang Z, Pascual-Anaya J, Zadissa A, Li W, Niimura Y, Huang Z, et al. The draft genomes of soft-shell turtle and green sea turtle yield insights into the development and evolution of the turtle-specific body plan. Nat Genet. 2013;45(6): 701-706.

3. Jarvis ED. Evolution of the pallium in birds and reptiles. In: Binder MD, Hirokawa N, Windhorst U, editors. Encyclopedia of Neuroscience. Springer Berlin Heidelberg; 2009. pp. 1390-1400.

4. Bruce LL, Neary TJ. The limbic system of tetrapods: A comparative analysis of cortical and amygdalar populations. Brain Behav Evol. 1995;46: 224-234.

5. Karten HJ. The organization of the avian telencephalon and some speculations on the phylogeny of the amniote telencephalon. In: Petras JN, Noback C, editors. Comparative and Evolutionary Aspects of the Vertebrate Central Nervous System. Vol. 167. Annals of the NewYork Academy of Science; New York: 1969. pp. 164-179.

6. Karten HJ. Homology and evolutionary origins of the 'neocortex'. Brain Behav Evol. 1991;38: 264-272.

7. Striedter GF. The telencephalon of tetrapods in evolution. Brain Behav Evol. 1997;49: 179-213.

8. Puelles L, Kuwana E, Puelles E, Bulfone A, Shimamura K, Keleher J, et al. Pallial and subpallial derivatives in the embryonic chick and mouse telencephalon, traced by the expression of the genes Dlx-2, Emx-1, Nkx-2.1, Pax-6, and Tbr-1. J Comp Neurol. 2000;424: 409-438.

9. Jarvis ED, Güntürkün O, Bruce L, Csillag A, Karten HJ, Kuenzel W, et al. Avian brains and a new understanding of vertebrate brain evolution. Nat Rev Neurosci. 2005;6: 151-159.

10. Butler AB, Reiner A, Karten HJ. Evolution of the amniote pallium and the origins of mammalian neocortex. Ann N Y Acad Sci. 2011;1225: 14-27.

11. Dugas-Ford J, Rowell JJ, Ragsdale CW. Cell-type homologies and the origins of the neocortex. Proc Natl Acad Sci U S A. 2012;109(42): 16974-16979.

12. Wagner GP. Homology, Genes, and Evolutionary Innovation. Princeton: Princeton University Press; 2014.

13. Visnapuu T, Plaas M, Reimets R, Raud S, Terasmaa A, Kõks S, et al. Evidence for impaired function of dopaminergic system in Wfs1-deficient mice. Behav Brain Res. 2013;244: 90-99.

14. Luuk H, Plaas M, Raud S, Innos J, Sütt S, Lasner H, et al. Wfs1-deficient mice display impaired behavioural adaptation in stressful environment. Behav Brain Res. 2009;198(2): 334-345.

15. Jayanthi S, McCoy MT, Beauvais G, Ladenheim B, Gilmore K, Wood W, et al. Methamphetamine induces dopamine D1 receptor-dependent endoplasmic reticulum stress-related molecular events in the rat striatum. PLoS One. 2009;4(6): e6092.

16. Cadet JL, Jayanthi S, McCoy MT, Beauvais G, Cai NS. Dopamine D1 receptors, regulation of gene expression in the brain, and neurodegeneration. CNS Neurol Disord Drug Targets 2010;9(5): 526-538.

17. Kawano J, Fujinaga R, Yamamoto-Hanada K, Oka Y, Tanizawa Y, Shinoda K. Wolfram syndrome 1 (Wfs1) mRNA expression in the normal mouse brain during postnatal development. Neurosci Res. 2009;64(2): 213-230.

18. Tekko T, Lilleväli K, Luuk H, Sütt S, Truu L, Örd T, et al. Initiation and developmental dynamics of Wfs1 expression in the context of neural differentiation and ER stress in mouse forebrain. Int J Dev Neurosci. 2014;35: 80-88.

19. Reiner A, Perkel DJ, Bruce L, Butler AB, Csillag A, Paxinos G, et al. Revised nomenclature for avian telencephalon and some related brainstem nuclei. J Comp Neurol. 2004;473: 377-414.

20. Sun Z and Reiner A. Localization of dopamine D1A and D1B receptor mRNAs in the forebrain and midbrain of the domestic chick. J Chem Neuroanat. 2000;19(4): 211-224.

21. Heimer L, Alheid GF, Olmos JS de, Groenenwegen HJ, Haber SN, Harlan RE, et al. The accumbens: beyond the core-shell dichotomy. J Neuropsychiatry Clin. Neurosci. 1997;9: 354-381.

22. Joel D and Weiner I. The connections of the dopaminergic system with the striatum in rats and primates: an analysis with respect to the functional and compartmental organization of the striatum. Neuroscience. 2000;96(3): 451-474.

23. Abellán A and Medina L. Subdivisions and derivatives of the chicken subpallium based on expression of LIM and other regulatory genes and markers of neuron subpopulations during development. J Comp Neurol. 2009;515: 465-501.

24. Veenman CL, Wild JM, Reiner A. Organization of the avian “corticostriatal” projection system: a retrograde and anterograde pathway tracing study in pigeons. J Comp Neurol. 1995;354: 87-126.

25. Mezey S and Csillag A. Selective striatal connections of midbrain dopaminergic nuclei in the chick (Gallus domesticus). Cell Tissue Res. 2002;308(1): 35-46.

26. Durstewitz D, Kröner S, Hemmings HC Jr, Güntürkün O. The dopaminergic innervation of the pigeon telencephalon: distribution of DARPP-32 and co-occurrence with glutamate decarboxylase and tyrosine hydroxylase. Neuroscience. 1998;83(3): 763-779.

27. Durstewitz D, Kröner S, Güntürkün O. The dopaminergic innervation of the avian telencephalon. Prog Neurobiol. 1999;59(2): 161-195.

28. Dietl MM and Palacios JM. Neurotransmitter receptors in the avian brain. I. Dopamine receptors. Brain Res. 1988;439: 354-359.

29. Ball GF, Casto JM, Balthazart J. Autoradiographic localization of D1-like dopamine receptors in the forebrain of male and female Japanese quail and their relationship with immunoreactive tyrosine hydroxylase. J Chem Neuroanat. 1995;9: 121-133.

30. Ouimet CC, Miller PE, Hemmings HC Jr, Walaas SI, Greengard P. DARPP-32, a dopamine- and adenosine 3':5'-monophosphate-regulated phosphoprotein enriched in dopamine-innervated brain regions. III. Immunocytochemical localization. J Neurosci. 1984;4(1): 111-124.

31. Ouimet CC, LaMantia AS, Goldman-Rakic P, Rakic P, Greengard P. Immunocytochemical localization of DARPP-32, a dopamine and cyclic-AMP-regulated phosphoprotein, in the primate brain. J Comp Neurol. 1992;323(2): 209-218.

32. Drago J, Gerfen CR, Lachowicz JE, Steiner H, Hollon TR, Love PS, et al. Altered striatal function in a mutant mouse lacking D1A dopamine receptors. Proc Natl Acad Sci U S A. 1994;91: 12564-12568.

33. Bálint E and Csillag A. Nucleus accumbens subregions: hodological and immunohistochemical study in the domestic chick (Gallus domesticus). Cell Tissue Res. 2007;327(2): 221-230.

34. Husband SA. Anatomy and function of the nucleus accumbens in the pigeon (Columba livia). Ph.D. Thesis. University of South Florida. 2004. Available: <http://scholarcommons.usf.edu/cgi/viewcontent.cgi?article=2086&context=etd>

35. Puelles L, Martínez-de-la-Torre M, Paxinos G, Watson CH, Martínez S. The Chick Brain in Stereotaxic Coordinates. An Atlas Featuring Neuromeric Subdivisions and Mammalian Homologues, 1st ed. San Diego: Academic Press, Elsevier; 2007.

36. Bardet SM, Cobos I, Puelles E, Martínez-De-La-Torre M, Puelles L. Chicken lateral septal organ and other circumventricular organs form in a striatal subdomain abutting the molecular striatopallidal border. J Comp Neurol. 2006;499(5): 745-767.

37. Luuk H, Koks S, Plaas M, Hannibal J, Rehfeld JF, Vasar E. Distribution of Wfs1 protein in the central nervous system of the mouse and its relation to clinical symptoms of the Wolfram syndrome. J Comp Neurol. 2008;509(6): 642-660.

38. Gabreëls BA, Swaab DF, de Kleijn DP. The vasopressin precursor is not processed in the hypothalamus of Wolfram syndrome patients with diabetes insipidus: evidence for the involvement of PC2 and 7B2. J Clin Endocrinol Metab. 1998;83(11): 4026-4033.

39. Moreno N and González A. The common organization of the amygdaloid complex in tetrapods: new concepts based on developmental, hodological and neurochemical data in anuran amphibians. Prog Neurobiol. 2006;78: 61-90.

40. LeDoux JE. Emotion circuits in the brain. Annu Rev Neurosci. 2000;23: 155-184.

41. Ehrlich I, Humeau Y, Grenier F, Ciocchi S, Herry C, Lüthi A. Amygdala inhibitory circuits and the control of fear memory. Neuron. 2009;62: 757-771.

42. Royer S, Martina M, Paré D. An inhibitory interface gates impulse traffic between the input and output stations of the amygdala. J Neurosci. 1999;19: 10575-10583.

43. Quirk GJ, Likhtik E, Pelletier JG, Paré D. Stimulation of medial prefrontal cortex decreases the responsiveness of central amygdala output neurons. J Neurosci. 2003;23: 8800-8807.

44. Marowsky A, Yanagawa Y, Obata K, Vogt KE. A specialized subclass of interneurons mediates dopaminergic facilitation of amygdala function. Neuron. 2005;48: 1025-1037.

45. Rosenkranz JA and Grace AA. Cellular mechanisms of infralimbic and prelimbic prefrontal cortical inhibition and dopaminergic modulation of basolateral amygdala neurons in vivo. J Neurosci. 2002;22: 324-337.

46. Fuxe K, Jacobsen KX, Hoistad M, Tinner B, Jansson A, Staines WA, et al. The dopamine D1 receptor-rich main and paracapsular intercalated nerve cell groups of the rat amygdala: relationship to the dopamine innervation. Neuroscience. 2003;119: 733-746.

47. Ulinski PS. Dorsal Ventricular Ridge: Treatise on Forebrain Organization in Reptiles and Birds. John Wiley & Sons Inc; 1983.

48. Tole S, Remedios R, Saha B, Stoykova A. Selective requirement of Pax6, but not Emx2, in the specification and development of several nuclei of the amygdaloid complex. J Neurosci. 2005;25(10): 2753-2760.

49. Atoji Y, Saito S, Wild JM. Fiber connections of the compact division of the posterior pallial amygdala and lateral part of the bed nucleus of the stria terminalis in the pigeon (Columba livia). J Comp Neurol. 2006;499: 161-182.

50. García-López M, Abellán A, Legaz I, Rubenstein JL, Puelles L, Medina L. Histogenetic compartments of the mouse centromedial and extended amygdala based on gene expression patterns during development. J Comp Neurol. 2008;506: 46-74.

51. Choi GB, Dong HW, Murphy AJ, Valenzuela DM, Yancopoulos GD, Swanson LW, et al. Lhx6 delineates a pathway mediating innate reproductive behaviors from the amygdala to the hypothalamus. Neuron. 2005;46: 647-660.

52. Bupesh M, Legaz I, Abellán A, Medina L. Multiple telencephalic and extratelencephalic embryonic domains contribute neurons to the medial extended amygdala. J Comp Neurol. 2011;519: 1505-1525.

53. Dielenberg RA, Hunt GE, McGregor IS. “When a rat smells a cat”: the distribution of Fos immunoreactivity in rat brain following exposure to a predatory odor. Neuroscience. 2001;104: 1085-1097.

54. Martinez RC, Carvalho-Netto EF, Ribeiro-Barbosa ER, Baldo MV, Canteras NS. Amygdalar roles during exposure to a live predator and to a predator-associated context. Neuroscience. 2011;172: 314-328.

55. Li CI, Maglinao TL, Takahashi LK. Medial amygdala modulation of predator odor-induced unconditioned fear in the rat. Behav Neurosci. 2004;118: 324-332.

56. Kõks S, Planken A, Luuk H, Vasar E. Cat odour exposure increases the expression of wolframin gene in the amygdaloid area of rat. Neurosci Lett. 2002;322(2): 116-120.

57. Steiger SS, Fidler AE, Valcu M, Kempenaers B. Avian olfactory receptor gene repertoires: evidence for a well-developed sense of smell in birds? Proc Biol Sci. 2008;75: 2309-2317.

58. Balthazart J and Taziaux M. The underestimated role of olfaction in avian reproduction? Behav Brain Res. 2009;200: 248-259.

59. Fluck E, Hogg S, Mabbutt PS, File SE. Behavioural and neurochemical responses of male and female chicks to cat odour. Pharmacol Biochem Behav. 1996;54: 85-91.

60. Bruce, LL. Evolution of the nervous system in reptiles. In: Kaas JH, editor. Evolutionary Neuroscience. Academic Press; 2009. pp. 233-264.

61. Swanson LW and Petrovich GD. What is the amygdala? Trends Neurosci. 1998;21: 323-331.

62. McDonald AJ. Is there an amygdala and how far does it extend? An anatomical perspective. Ann N Y Acad Sci. 2003;985: 1-21.

63. Martínez-García F, Novejarque A, Lanuza E. The evolution of the amygdala in vertebrates. In: Kaas JH, editor. Evolutionary Neuroscience. Academic Press; 2009. pp. 313-392.

64. Zeier H and Karten HJ. The archistriatum of the pigeon: organization of afferent and efferent connections. Brain Res. 1971;31: 313-326.

65. Reiner A, Yamamoto K, Karten HJ. Organization and evolution of the avian forebrain. Anat Rec A Discov Mol Cell Evol Biol. 2005;287(1): 1080-1102.

66. Atoji Y, Wild JM, Yamamoto Y, Suzuki Y. Intratelencephalic connections of the hippocampus in pigeons (Columba livia). J Comp Neurol. 2002;447: 177-199.
