# Supplementary figures and images for: Wfs1 is expressed in dopaminoceptive regions of the amniote brain and modulates levels of D1-like receptors
Source: PLoS One. 2017 Mar 7;12(3):e0172825. doi: 10.1371/journal.pone.0172825 (PMC5436468; doi:10.1371/journal.pone.0172825)

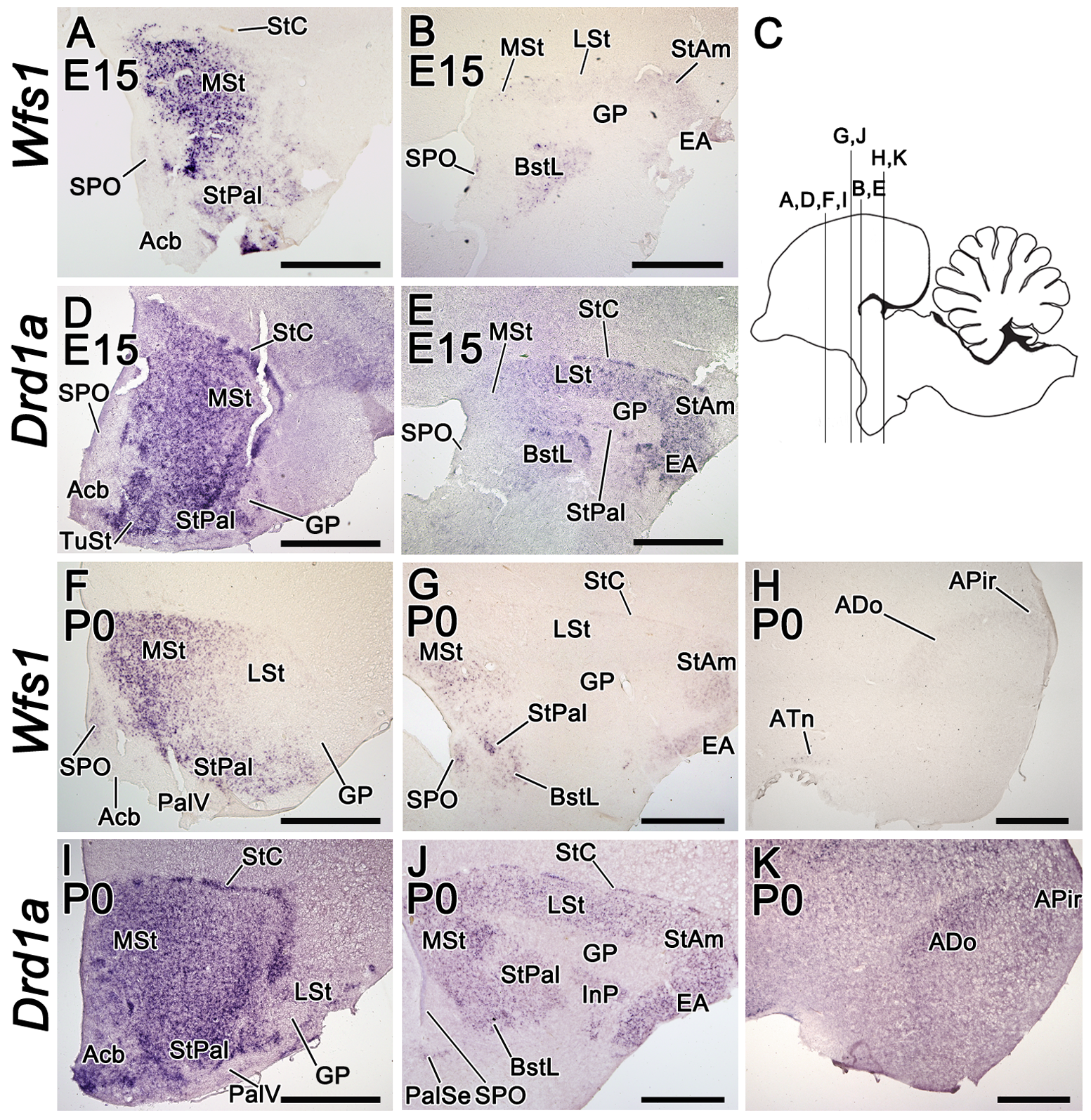

Supplement: S1 Fig — Medial side of the sections is on the left and lateral side on the right. The section plane is shown in image C. The probes are indicated on the left side of the figure and stages are indicated in the images. Note that in the lateral part of MSt and in anterior LSt, Drd1a is present, but not Wfs1 (compare A to D and F to I). In SPO, Wfs1 is expressed, but not Drd1a (compare A to D, B to E, F to I, G to J). In Acb, Drd1a is expressed, but not Wfs1 (compare A to D, F to I). For abbreviations, see list. Scale bar is 1mm. (TIF) [file pone.0172825.s003.tif]

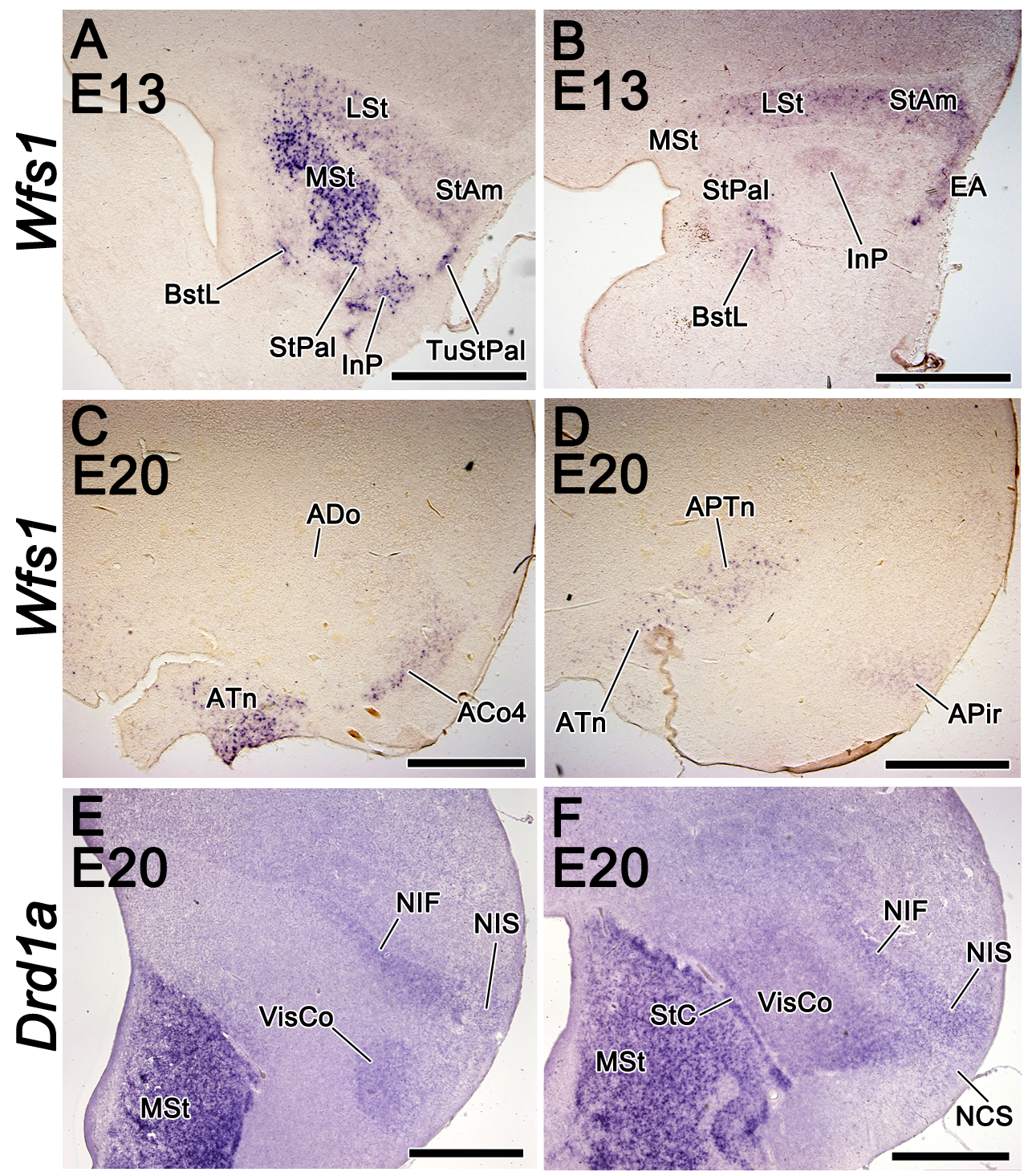

Supplement: S2 Fig — Medial side of the sections is on the left and lateral side on the right. The probes are indicated on the left and stages are indicated on the images. By E13, most of the subpallial regions were expressing Wfs1 (A,B). In pallial amygdala, the expression of Wfs1 was most widespread at E20 (C,D). Several regions of the nidopallium were expressing Drd1a in developing brain (E,F). For abbreviations, see list. Scale bar is 1mm. (TIF) [file pone.0172825.s004.tif]

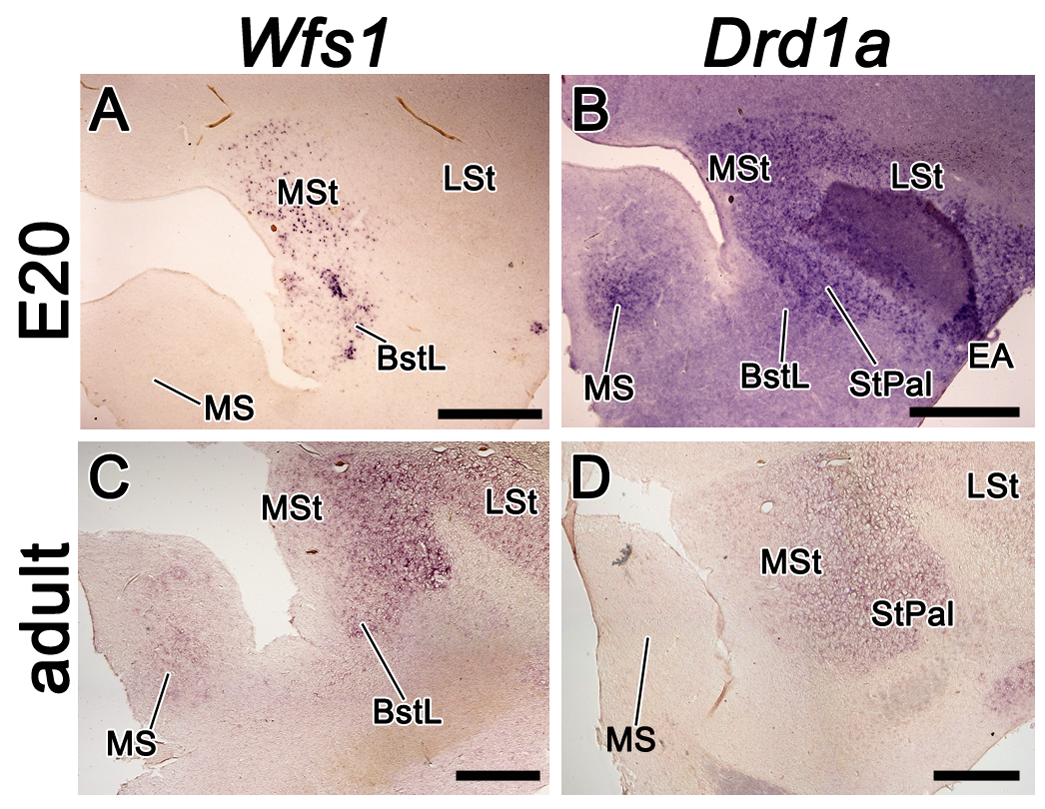

Supplement: S3 Fig — Medial side of the sections is on the left and the lateral side on the right. The probes are indicated on the top and stages are indicated on the left. Note that during the development, only Drd1a is present in MS (compare A and B), but in adulthood, only Wfs1 is present in the same structure (compare C and D). For abbreviations, see list. Scale bar is 1mm. (TIF) [file pone.0172825.s005.tif]
