# Supplementary material for: Wfs1 is expressed in dopaminoceptive regions of the amniote brain and modulates levels of D1-like receptors
Source: PLoS One. 2017 Mar 7;12(3):e0172825. doi: 10.1371/journal.pone.0172825 (PMC5436468; doi:10.1371/journal.pone.0172825)
Supplement: S1 Table — The binding of 4 nM [3H]SCH23390 was determined in duplicates or triplicates in the absence (for total binding) or in the presence (for nonspecific binding) of 10 μM (+)-butaclamole at tissue concentration 6.7 mg/ml. The specific binding was calculated as difference between total and nonspecific bindings and presented as mean value for particular mouse. (DOCX) [file pone.0172825.s006.docx]

**S1 Table**

**The number of binding sites of D1-like receptors in hippocampal membranes of wt and *Wfs1* knockout mice.** The binding of 4 nM [^3^H]SCH23390 was determined in duplicates or triplicates in the absence (for total binding) or in the presence (for nonspecific binding) of 10 μM (+)-butaclamole at tissue concentration 6.7 mg/ml. The specific binding was calculated as difference between total and nonspecific bindings and presented as mean value for particular mouse.

| **Summary**  [^3^H]SCH23390_BOUND_, fmol/mg tissue | | |
| --- | --- | --- |
|  | **wt** | ***Wfs1 -/-*** |
| Number of mice | 24 | 22 |
| Mean | 2,81 | 1,42 |
| SEM | 0,53 | 0,28 |
| **Unpaired two-tailed *t* test** | | |
| Difference between means | 1,39 ± 0,61 | |
| P value | 0,0283 | |
| Are means signif. different? (P < 0.05) | Yes | |
| t, df | t=2,268 df=44 | |
| **Data of individual mice** [^3^H]SCH23390_BOUND_, fmol/mg tissue | | |
|  | **wt** | ***Wfs1 -/-*** |
|  | 5,37 | 1,42 |
|  | 4,05 | 1,43 |
|  | 8,48 | 2,93 |
|  | 0,22 | 3,31 |
|  | 6,05 | 0,21 |
|  | 1,74 | 0,68 |
|  | 5,26 | 2,03 |
|  | 1,18 | 0,00 |
|  | 2,04 | 3,31 |
|  | 1,60 | 3,84 |
|  | 1,21 | 4,26 |
|  | 4,56 | 0,00 |
|  | 0,20 | 0,71 |
|  | 2,30 | 0,71 |
|  | 0,23 | 0,25 |
|  | 1,90 | 1,81 |
|  | 0,27 | 0,22 |
|  | 0,00 | 0,017 |
|  | 0,34 | 0,27 |
|  | 0,76 | 1,57 |
|  | 7,48 | 1,33 |
|  | 2,65 | 0,89 |
|  | 2,63 | - |
|  | 6,98 | - |
